# Validated inference of smoking habits from blood with a finite DNA methylation marker set

**DOI:** 10.1007/s10654-019-00555-w

**Published:** 2019-09-07

**Authors:** Silvana C. E. Maas, Athina Vidaki, Rory Wilson, Alexander Teumer, Fan Liu, Joyce B. J. van Meurs, André G. Uitterlinden, Dorret I. Boomsma, Eco J. C. de Geus, Gonneke Willemsen, Jenny van Dongen, Carla J. H. van der Kallen, P. Eline Slagboom, Marian Beekman, Diana van Heemst, Leonard H. van den Berg, BIOS Consortium, Liesbeth Duijts, Vincent W. V. Jaddoe, Karl-Heinz Ladwig, Sonja Kunze, Annette Peters, M. Arfan Ikram, Hans J. Grabe, Janine F. Felix, Melanie Waldenberger, Oscar H. Franco, Mohsen Ghanbari, Manfred Kayser

**Affiliations:** 1 Department of Epidemiology, Erasmus MC University Medical Center Rotterdam, Dr. Molewaterplein 40, 3015 GD Rotterdam, The Netherlands; 2 Department of Genetic Identification, Erasmus MC University Medical Center Rotterdam, Dr. Molewaterplein 40, 3015 GD Rotterdam, The Netherlands; 3 Research Unit of Molecular Epidemiology, Helmholtz Zentrum München, German Research Center for Environmental Health (GmbH), Ingolstädter Landstr. 1, 85764 Neuherberg, Germany; 4 Institute of Epidemiology, Helmholtz Zentrum München, German Research Center for Environmental Health (GmbH), Ingolstädter Landstr. 1, 85764 Neuherberg, Germany; 5 Institute for Community Medicine, University Medicine Greifswald, Walther-Rathenau-Str. 48, 17475 Greifswald, Germany; 6 German Center for Cardiovascular Research (DZHK), Partner Site Greifswald, 17475 Greifswald, Germany; 7 Key Laboratory of Genomic and Precision Medicine, Beijing Institute of Genomics, Chinese Academy of Sciences, NO.1 Beichen West Road, Chaoyang District, 100101 Beijing, People’s Republic of China; 8 University of Chinese Academy of Sciences, No.19A Yuquan Road, Shijingshan District, 100049 Beijing, People’s Republic of China; 9 Department of Internal Medicine, Erasmus MC University Medical Center Rotterdam, Dr. Molewaterplein 40, 3015 GD Rotterdam, The Netherlands; 10 Department of Biological Psychology, Vrije Universiteit, Van der Boechorststraat 7-9, 1081 BT Amsterdam, The Netherlands; 11 Department of Internal Medicine, Maastricht University Medical Center, Randwycksingel 35, 6229 EG Maastricht, The Netherlands; 12 Cardiovascular Research Institute Maastricht (CARIM), Maastricht University, Universiteitssingel 50, 6229 ER Maastricht, The Netherlands; 13 Molecular Epidemiology, Department of Biomedical Data Sciences, Leiden University Medical Center, P.O. box 9600, 2300 RC Leiden, The Netherlands; 14 Gerontology and Geriatrics, Department of Internal Medicine, Leiden University Medical Center, P.O. box 9600, 2300 RC Leiden, The Netherlands; 15 Department of Neurology, Brain Center Rudolf Magnus, University Medical Center Utrecht, Postbus 85500, 3508 GA Utrecht, The Netherlands; 16 Division of Respiratory Medicine and Allergology and Division of Neonatology, Department of Pediatrics, Erasmus MC University Medical Center Rotterdam, Dr. Molewaterplein 40, 3015 GD Rotterdam, The Netherlands; 17 The Generation R Study Group, Erasmus MC University Medical Center Rotterdam, Dr. Molewaterplein 40, 3015 GD Rotterdam, The Netherlands; 18 Department of Pediatrics, Erasmus MC University Medical Center Rotterdam, Dr. Molewaterplein 40, 3015 GD Rotterdam, The Netherlands; 19 German Center for Cardiovascular Research (DZHK), Partner Site Munich Heart Alliance, 80802 Munich, Germany; 20 Institute for Medical Informatics, Biometrics and Epidemiology, Ludwig-Maximilians-Universität (LMU) Munich, Marchioninistr. 15, 81377 Munich, Germany; 21 Department of Psychiatry and Psychotherapy, University Medicine Greifswald, Ellernholzstraße 1-2, 17475 Greifswald, Germany; 22 Department of Genetics, School of Medicine, Mashhad University of Medical Science, PO Box 91735-951, 9133913716 Mashhad, Iran

**Keywords:** Epigenetics, DNA methylation, Smoking inference, Epidemiology, Forensics

## Abstract

Inferring a person’s smoking habit and history from blood is relevant for complementing or replacing self-reports in epidemiological and public health research, and for forensic applications. However, a finite DNA methylation marker set and a validated statistical model based on a large dataset are not yet available. Employing 14 epigenome-wide association studies for marker discovery, and using data from six population-based cohorts (N = 3764) for model building, we identified 13 CpGs most suitable for inferring smoking versus non-smoking status from blood with a cumulative Area Under the Curve (AUC) of 0.901. Internal fivefold cross-validation yielded an average AUC of 0.897 ± 0.137, while external model validation in an independent population-based cohort (N = 1608) achieved an AUC of 0.911. These 13 CpGs also provided accurate inference of current (average AUC_crossvalidation_ 0.925 ± 0.021, AUC_externalvalidation_0.914), former (0.766 ± 0.023, 0.699) and never smoking (0.830 ± 0.019, 0.781) status, allowed inferring pack-years in current smokers (10 pack-years 0.800 ± 0.068, 0.796; 15 pack-years 0.767 ± 0.102, 0.752) and inferring smoking cessation time in former smokers (5 years 0.774 ± 0.024, 0.760; 10 years 0.766 ± 0.033, 0.764; 15 years 0.767 ± 0.020, 0.754). Model application to children revealed highly accurate inference of the true non-smoking status (6 years of age: accuracy 0.994, N = 355; 10 years: 0.994, N = 309), suggesting prenatal and passive smoking exposure having no impact on model applications in adults. The finite set of DNA methylation markers allow accurate inference of smoking habit, with comparable accuracy as plasma cotinine use, and smoking history from blood, which we envision becoming useful in epidemiology and public health research, and in medical and forensic applications.

**Electronic supplementary material:**

The online version of this article (10.1007/s10654-019-00555-w) contains supplementary material, which is available to authorized users.

## Introduction

Several studies suggest that tobacco smoking impacts the human epigenome, particularly by changing DNA methylation patterns [1, 2]. DNA methylation is catalyzed by DNA methyltransferases (DNMT’s); the carcinogens in cigarette smoke cause double-strand DNA breaks and the DNA repair sites recruit DNMT1 [3], which methylates cytosines in CpGs adjacent to the repaired nucleotides [4]. Nicotine was shown to down-regulate DNMT1, and mRNA and protein expression [5]. Furthermore, cigarette smoke condensate increases expression of Sp1, a transcription factor that binds to GC-rich motifs in gene promoters, preventing de novo methylation [6–9]. In recent years, various epigenome-wide association studies (EWASs) have provided a long list of CpGs significantly associated with tobacco smoking habits in blood [10]. Although there are strong smoking associations across the epigenome, some studies suggest that after smoking cessation, DNA methylation patterns can return back to those found in never smokers [11, 12].

Smoking is a well-known risk factor for the development of several diseases [13, 14]. Therefore, studies that investigate smoking and its effect on mortality and morbidity rely on accurate assessments of smoking exposure. These studies use mainly self-reported smoking questionnaires to collect this information, which could result in underestimation and misrepresent the degree of the true smoking exposure [15]. In particular, it is possible that specific groups of participants, for instance pregnant women, are more reluctant to confide that they smoke [16]. Hence, the ability to reliably and accurately infer a person’s smoking habit from blood is relevant in epidemiology and public health research as well as in medical practice, because such an approach could complement, or even replace, self-reported smoking questionnaires.

Moreover, inference of a person’s smoking habit from blood traces found at crime scenes would allow the broadening of DNA investigative intelligence beyond the currently considered parameters of appearance, bio-geographic ancestry and age, thus helping to better find unknown perpetrators of crime who are not identifiable via standard forensic DNA profiling [17]. Blood-based toxicological tests for measurement of tobacco exposure exist; however, they assess current and acute, rather than habitual, smoking [18]. In addition, biomarkers used include nicotine itself or its metabolite cotinine, and their accurate detection of current smokers is affected by their short half-lives (2–3 h vs. 15–19 h for nicotine and cotinine, respectively) and individual variation in metabolic rates [19]. Therefore, when using the cotinine-based approach false-negatives can be easily obtained, and also false-positives may occur in former smokers that use nicotine replacement therapy [20]. Given these constrains of current toxicology blood measures, and considering the recent progress in understanding the impact of smoking on epigenetic variation, we envision DNA methylation from blood as a promising approach for long-term habitual smoking behaviour.

Although progress has been made in understanding the epigenetic impact of smoking [1], only a limited number of studies have explored the inference of smoking habits from blood with DNA methylation markers, albeit with various limitations such as small sample size, limited validation, restricting to smokers and non-smokers and not considering former smokers in the model building, and/or utilizing large numbers of CpGs [21–27]. Reliable studies on the validated inference of a person’s smoking habits and history from blood with a finite set of DNA methylation markers and based on statistical models with large underlying data are not available as of yet. A finite number of DNA methylation markers achieving maximal prediction accuracy would be especially beneficial for those practical applications where—due to limited DNA quality and quantity, a common problem in forensics—it is impossible to apply standard DNA methylation microarray technology [17].

With this study, we aimed to identify a robust, finite set of DNA methylation markers in blood and, based on this finite biomarker set, develop accurate, reliable and validated statistical models for inferring a person’s tobacco smoking habits and history from blood, which we envision becoming useful in future epidemiology and public health research as well as medical and forensic applications.

## Materials and methods

### Study population

This study was embedded within the Biobank-based Integrative Omics Study (BIOS) Consortium [28], which consists of six Dutch cohorts (N = 3118), including the Rotterdam Study (RS) (N = 584) [29], Cohort on Diabetes and Atherosclerosis Maastricht (CODAM) (N = 156) [30], The Netherlands Twin Register (NTR) (N = 894) [31], Leiden Longevity Study (LLS) (N = 625) [32], Prospective ALS Study Netherlands (PAN) (N = 167) [33] and LifeLines (LL) (N = 692) [34]. Additionally, we included another 646 unrelated participants from the Rotterdam Study (RS-III-1) not included in BIOS. We externally validated our model in the Kooperative Gesundheitsforschung in der Region Augsburg (KORA) study (F4, N = 1608) [35], as well as in the Study of Health in Pomerania (SHIP)-Trend (N = 244) [36] cohort. Characteristics of all cohorts used can be found in Online Resource 1: Table S1. We additionally tested our model in samples from children included in the Generation R Study [37], in particular, we used data from children participating at birth (N = 1111), at the age of 6 years (N = 355), and at the age of 10 years (N = 309), of which 197 overlapped between all three time points, providing longitudinal data (Online Resource 1: Table S2). The smoking status information was obtained using questionnaires. The study characteristics are described in detail in Online Resource 2: Supplemental methods.

### DNA methylation quantification

DNA was extracted from whole peripheral blood in all studies using standard procedures. All studies used the Illumina Infinium Human Methylation 450 K BeadChip (Illumina Inc, San Diego, CA, USA) for epigenome-wide DNA methylation measurements, except the SHIP-Trend study, which used the more recent Infinium MethylationEPIC BeadChip (Illumina Inc, San Diego, CA, USA). DNA methylation data pre-processing for cohorts included in the BIOS consortium were conducted together via the pipeline created by Tobi et al. [38, 39]. The DNA methylation data pre-processing in the external validation cohorts and the Generation R Study were done independently. The methylation proportion of a CpG site was reported as a methylation β-value in the range of 0 (representing completely non-methylated sites) to 1 (representing completely methylated sites). Further study-specific methods can be found in Online Resource 2: Supplemental methods.

### Ascertainment of smoking-associated CpGs

EWASs using the Illumina Infinium Human Methylation 27 K or 450 K BeadChip investigating smoking-induced changes in DNA methylation patterns were reviewed [2, 21, 40–50]. We excluded studies [11] that used cohorts included in our model-building dataset, to avoid over-estimation of our model. Envisioning future laboratory tool development, we only selected robust CpGs that were (1) highlighted in two or more studies, (2) with at least 10% difference in mean or median (depending on availability per EWAS) β-values between current smokers and never-smokers (or non-smokers when non-smoking data was available) in at least one of the studies, and (3) with the same direction in β-value difference between current smokers and never/non-smokers in all studies investigated.

### Statistical modeling for current smoking habits

Of the total participants considered for model building (N_total_ = 5178), we excluded those with (1) missing data for smoking habits (1206 participants), (2) missing β-values for the predictive CpGs (82 participants), or (3) extreme outliers for one or more CpGs (mean ± 4 SD) (126 participants). In the end, we included 3764 participants in the final model building set, who were then categorized based on their smoking habits as (1) current smokers or (2) former and never smokers combined. The association between the candidate CpGs and smoking habits (smokers vs. non-smokers) was replicated in our model building dataset using binomial regression analysis adjusted for age and sex using the “glm” function with “binomial” as family and “logit” as link. To identify the most informative set of DNA methylation predictors from the candidate CpGs, the association between the complete set of predictive CpGs and smoking habits was assessed in a binary logistic regression analysis, using the “glm” function with “binomial” as family and “logit” as link. Backward elimination procedures were used for the marker selection process. We excluded the CpGs one by one based on their absolute z-statistic per regression (calculated by dividing the regression coefficient by its standard error) assessed using the “VarImp” function (r-package “caret”). The predictive CpG with the lowest absolute z-statistic in the regression was removed. The model was applied to the dataset with the “predict” function (type = “response”) and the confusion matrix (r-package “caret”) was conducted using a probability threshold of 0.5. The prediction performance of the model was additionally assessed using “prediction” and “performance” (r-package “ROCR”), the Area Under the Curve (AUC) per model was calculated (r-package “ROCR”) and a cumulative AUC profile was conducted for each model to obtain a cumulative AUC profile. We selected the best-fit prediction model using a combination of the backward elimination approach and the Chi squared test. In particular, we compared the model including all CpGs (model_FULL_) with the model excluding one CpGs, (model_FULL-1CpG_), this model _FULL-1CpG_ was then compared with the model excluding another CpG (model_FULL-2CpGs_), following the same order as conducted via the backward approach, and so on until we noticed a statistically significant difference between two models in the backward approach. Subsequently, we tested the inclusion of age, sex and cell counts to the final model.

### Former smokers as additional category

Participants included in the model building dataset (N = 3764) without additional smoking data, including the age someone stopped smoking (former smokers) or the age someone started smoking or the number of cigarettes someone smokes per day (current smokers), were excluded, resulting in a dataset including 2939 participants. The association between the previously selected predictive CpGs and the three smoking categories was assessed in a multinomial regression analysis, using the “multinom” function (r-package “nnet”). We predicted the smoking categories using the “predict” function (type = “class”) and the confusion matrix (r-package “caret”) was conducted. The AUC per category was conducted using the “predict” function (type = “probs”) and “roc” function (r-package “pROC”).

### Smoking cessation time inference in former smokers

In the former smokers (N = 1332), smoking cessation time was calculated as one’s age minus the age one stopped smoking. The participants were split into two categories for three models. For model 1, ≥ 5 years cessation time were coded as “1” and < 5 years smoking cessation were coded as “0”, for model 2, ≥ 10 years cessation time were coded as “1” and < 10 years smoking cessation were coded as “0”, and for model 3, ≥ 15 years cessation time were coded as “1” and < 15 years smoking cessation were coded as “0”. The predictions were conducted using the same method as described for the current versus non-smokers model. Probability thresholds were set to 0.8733, 0.7650 and 0.6397 respectively.

### Pack-year inference in current smokers

For the current smokers (N = 364) the pack-years were calculated as the number of cigarettes smoked per day divided by 20, multiplied by the total years of smoking. The participants were categorized into two categories for two models. For model 1, ≥ 15 pack-years were coded as “1” and < 15 pack-years coded as “0”, for model 2, ≥ 10 pack-years were coded as “1” and < 10 pack-years coded as “0”. The predictions were conducted using the same method as described for the current *vs* non-smokers model.

### Pack-years (current-), smoking cessation time (former-) and never smokers

We combined the pack-year inference in current smokers with the cessation time in former and never smokers, resulting into five categories in two models (N = 2939) for inferring life-time smoking information. For model 1, the current smokers ≥ 15 pack-years were coded as “5”, with < 15 pack-years were coded as “4”, the former smokers ≤ 10 years smoking cessation were coded as “3”, with > 10 years smoking cessation were coded as “2” and never smokers were coded as “1”. In the second model the same categories were used except for the pack-years which were now divided in ≥ 10 pack-years (coded as “5”) and < 10 pack-years (coded as “4”). The predictions were conducted using the same method as described for the current *vs* former *vs* never smokers model.

### Internal validation of the developed prediction models

For internal validation of the developed predictive models, we adopted a fivefold cross-validation scheme [51], in which the whole dataset is first randomly distributed into five equal and non-overlapping subsets. Four of the subsets (80% of the data) are combined to form a dataset used to train the logistic regression model which is then tested by inferring the smoking habits in the remaining dataset (20% of the data). This resulted in five different training (80%) and testing (20%) sets. The model was trained in the five training sets and applied to corresponding testing sets, resulting in five logistic regression models. Subsequently, we used the bootstrap method (r-packages “boot” and “parallel”) as additional internal validation to correct for potential overestimation of the prediction, since we use the same data for model building and predictions. We generated 1000 bootstrap samples, with replacement from the dataset for which we estimated the model and applied each fitted model to the original sample, resulting in 1000 AUC estimates. Thereafter, we recalculated the prediction accuracy by applying the fitted model to the bootstrap sample itself. The performance in the bootstrap sample represents an estimation of the apparent performance, and the performance in the original sample represents test performance. The difference between the average of the two conducted AUCs is a stable estimate of the optimism. We corrected for prediction overestimation by subtracting the optimism from the apparent AUC, to obtain an improved estimate of the prediction AUC [52, 53].

### External validation of the developed prediction models

We externally validated our prediction models in two independent cohorts from German-European origin. The full models were validated in the KORA F4 study (N = 1608). Additionally, we externally validated our models in the SHIP-Trend study (N = 244). In this cohort, the EPIC methylation array was used which does not include all CpGs of the 450 K array. We therefore first generated the prediction models based on the overlapping CpGs in the model building dataset and subsequently externally validated them in the SHIP-Trend dataset.

### Comparing performance of CpG-based model with cotinine level cut-off

We compared the outcomes of the CpG model to infer current *vs* non-smokers with the outcomes using a cotinine level cut-off of 50 ng/mL [54, 55] and applied smoking information from self-reports as reference. We employed a subset of our model building dataset (N = 488 participants included in NTR [56]) in which both DNA methylation levels and cotinine levels were available. First, participants were categorized as smokers when their plasma cotinine levels were > 50 ng/mL, or as non-smokers with cotinine levels ≤ 50 ng/mL, threshold according to previous studies including the used cotinine data [54, 55]. Second, the current versus non-smokers CpG model was applied to this subset, obtaining the inferred smoking status for the participants. Third, we compared the obtained smoking status for both models with the information obtained from the self-reported questionnaires and computed the sensitivity and specificity per model.

### Application of the developed prediction model in newborns and young children

Studies have shown the impact of prenatal smoking exposure on the DNA methylation pattern of the offspring [57] and the ability of predicting maternal smoking status using these patterns [58]. In this context, we wanted to test the effect of prenatal exposure on model application in adults. Hence, when an adult does not smoke, but was exposed to prenatal smoking, do we predict this person indeed as a true non-smoker? To test for this putative impact of exposure to prenatal smoking on epigenetic inference of smoking habits using our model, we tested our model in umbilical cord blood of newborns (N = 1111), and in whole blood of children at the ages of six (N = 355) and 10 years (N = 309). We used five different analyses to evaluate the effects of active smoking of the mothers and passive smoking of the mothers (i.e. smoking of others in the mother’s home and work environment) during pregnancy on smoking habit inference using our model. In our first analysis, we did not take the smoking habits of the pregnant mothers or others in the pregnant mother’s home and work environment into account and all children were coded as non-smokers. The proportion of accurately predicted cases was calculated using a probability threshold of 0.5. In each of the following analyses, we coded the children “1” if their parents met the smoking habit criteria, otherwise they were coded as “0”. So, in the second analysis, only sustained maternal smoking throughout pregnancy was considered. Therefore, the children of mothers that smoked during the whole pregnancy were coded as “1”. In the third analysis, we additionally included the children of mothers who stopped smoking when they realized that they were pregnant by coding these children as “1”. In the fourth analysis, we additionally included smoking of the father and/or others in the mother’s household/at work (> 1 h per day) during pregnancy (i.e. passive smoking). In the fifth analysis, we assessed the sole effect of passive smoking i.e., where the mother did not smoke but the father or someone else in the house or at work (> 1 h per day) smoked during the pregnancy of the mother. For 197 children, DNA methylation levels were measured at all three time points, i.e. birth, 6 years of age and 10 years of age; hence, we repeated the previous models again in these children to allow a direct comparison of the findings at these three time points in the same individuals.

## Results

### Ascertaining candidate DNA methylation markers for inferring smoking habits from blood

**Fig. 1:**
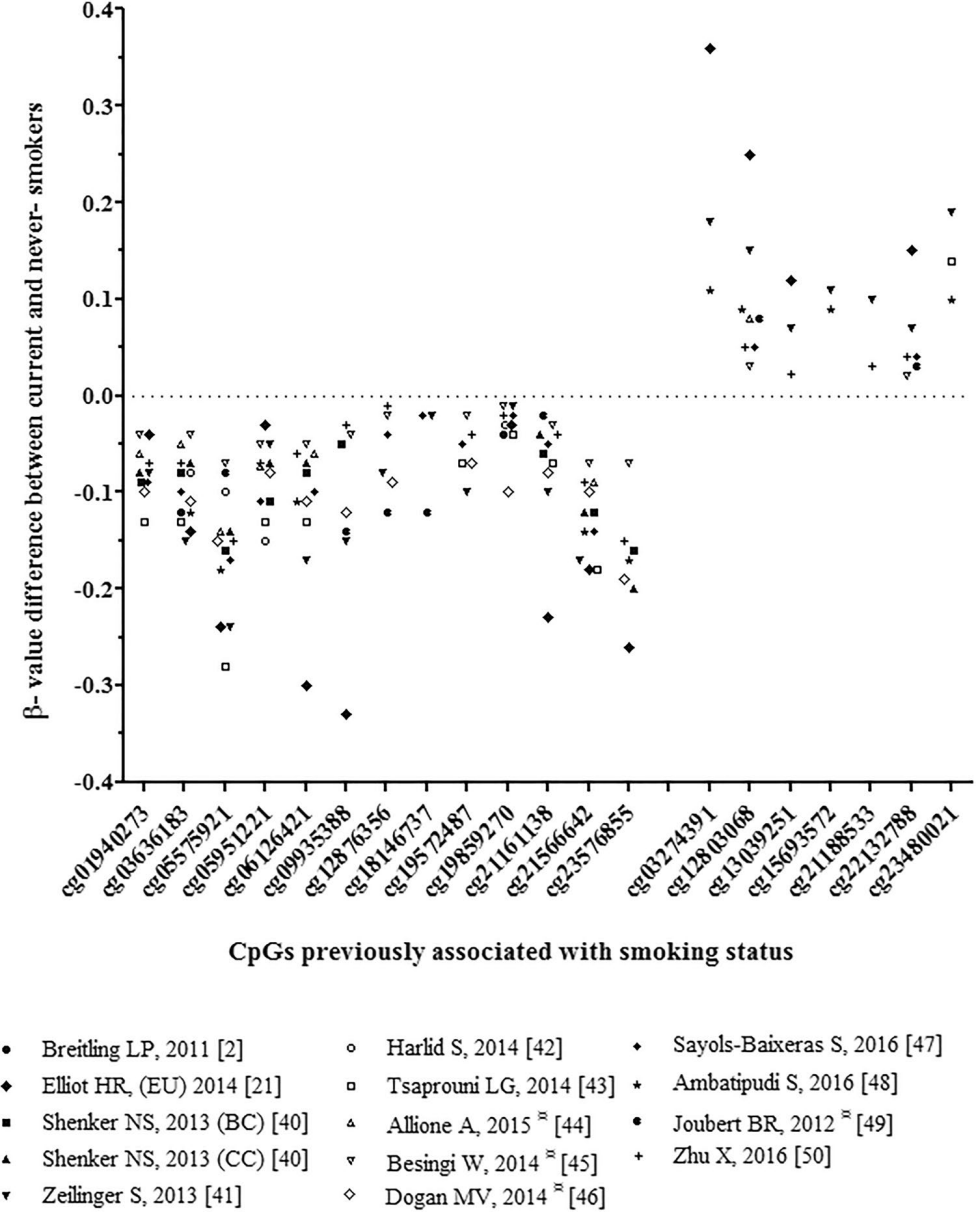
**DNA methylation β-value differences between smokers and never-smokers for the top 20 smoking-associated CpGs.** Previously reported differences in β-values in mean or median (depending on availability per EWAS) between smokers and never-smokers (^¤^ or non-smokers, when non-smoking data was available) for the selected 20 top-associated CpGs obtained from the 14 reviewed EWASs on smoking habits that did not include samples used here for model building

We inspected 14 published EWASs on tobacco smoking habits (N_total_ = 7015) [2, 21, 40–50] to identify smoking-associated CpGs as candidate DNA methylation markers for prediction modeling of smoking habits. CpGs were selected as candidate prediction markers if they met three criteria as mentioned in the method section. This procedure highlighted 20 top smoking-associated CpGs as candidate markers used for further analyses (Table 1). The differences in β-values between smokers and never-/non-smokers reported previously for these 20 top smoking-associated CpGs are illustrated in Fig. 1.

**Table 1:** Top 20 smoking-associated CpGs from 14 previous EWASs considered here for marker sub-selection and their contribution to smoking inference from blood

CpG ID	Chr:position^b^	Gene ID^c^	Location of CpG	Cumulative AUC
cg05575921^a^	5:373,378	*AHRR*	Gene body	0.8801
cg13039251^a^	5:32,018,601	*PDZD2*	Gene body	0.8888
cg03636183^a^	19:17,000,585	*F2RL3*	Gene body	0.8883
cg12803068^a^	7:45,002,919	*MYO1G*	Gene body	0.8889
cg22132788^a^	7:45,002,486	*MYO1G*	Gene body	0.8934
cg06126421^a^	6:30,720,080	NA	–	0.8929
cg21566642^a^	2:233,284,661	NA	–	0.8957
cg23576855^a^	5:373,299	*AHRR*	Gene body	0.8967
cg15693572^a^	3:22,412,385	NA	–	0.8982
cg05951221^a^	2:233,284,402	NA	–	0.8989
cg01940273^a^	2:233,284,934	NA	–	0.8998
cg12876356^a^	1:92,946,825	*GFI1*	Gene body	0.9005
cg09935388^a^	1:92,947,588	*GFI1*	Gene body	0.9010
cg19572487	17:38,476,024	*RARA*	5′UTR	0.9012
cg19859270	3:98,251,294	*GPR15*	Gene body (1st Exon)	0.9015
cg18146737	1:92,946,700	*GFI1*	Gene body	0.9015
cg21161138	5:399,360	*AHRR*	Gene body	0.9015
cg23480021	3:22,412,746	NA	–	0.9016
cg21188533	3:53,700,263	*CACNA1D*	Gene body	0.9015
cg03274391	3:22,413,232	NA	–	0.9015

*NA* not annotated to any gene according to the Illumina Infinium Human Methylation 450 K annotation file

*AUC* Area under the curve

^a^CpGs included in our final 13 CpG-model

^b^Genome coordinates provided by Illumina (GRCh37/hg19)

^c^According to the Illumina Infinium Human Methylation 450 K annotation file

### Building CpG-based models for inferring smoking habit and history from blood

**Fig. 2:**
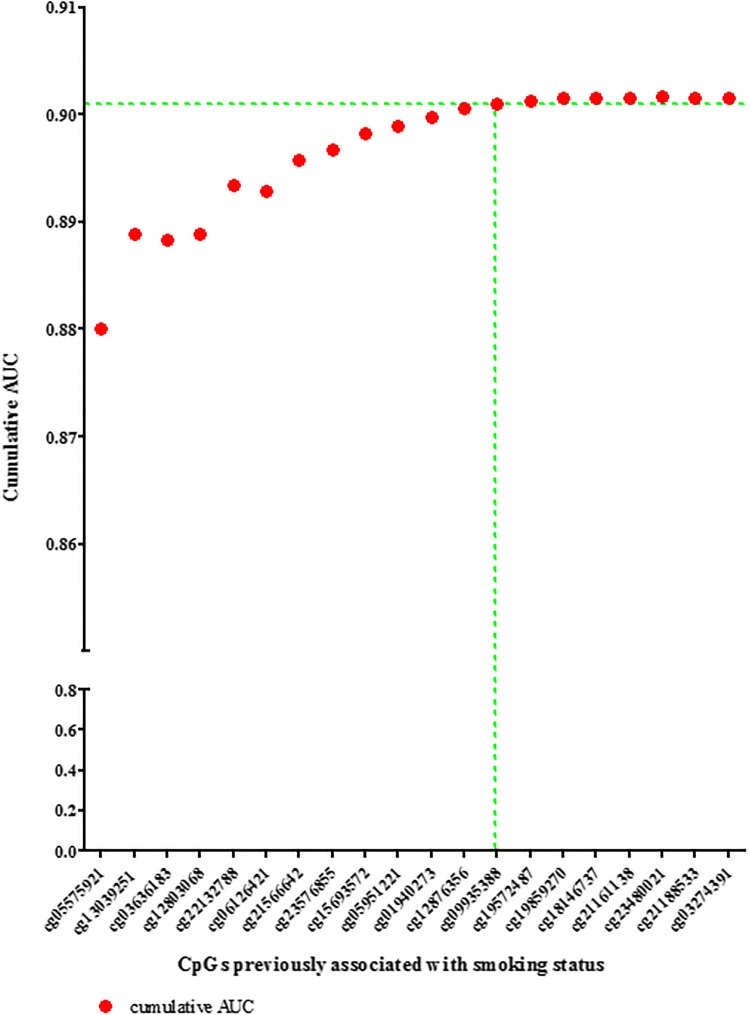
**Cumulative AUC profile for smoking habit inference from blood based on the top 20 CpGs.** The 20 CpGs were selected from previous EWASs on smoking habits (see Fig. 1) and were tested in the model-building set (N = 3764). Presented is the cumulative contribution of each of the selected 20 CpGs to the model-based smoking habit inference, shown as the AUC plotted against the number of CpGs included in the binary logistic regression model. In the model selection process, first all CpGs were included, and using backward elimination procedures, those with the lowest z-statistic per model were removed one by one. After 13 CpGs, the AUC plateaus; therefore, and by considering the results from Chi squared testing, these 13 CpGs were used for further analyses

Following the replication of the association between the CpGs and smoking habits (smokers vs. non-smokers) after adjusting for age and sex (Online source Table 3), we assessed the predictive effect of the selected 20 candidate markers in the model building dataset (N = 3764). Starting with a model including all 20 CpGs, the CpG with the lowest z-value per model was sequentially removed, and the AUC was calculated for each model to obtain a cumulative AUC profile (Table 1; Fig. 2).

**Fig. 3:**
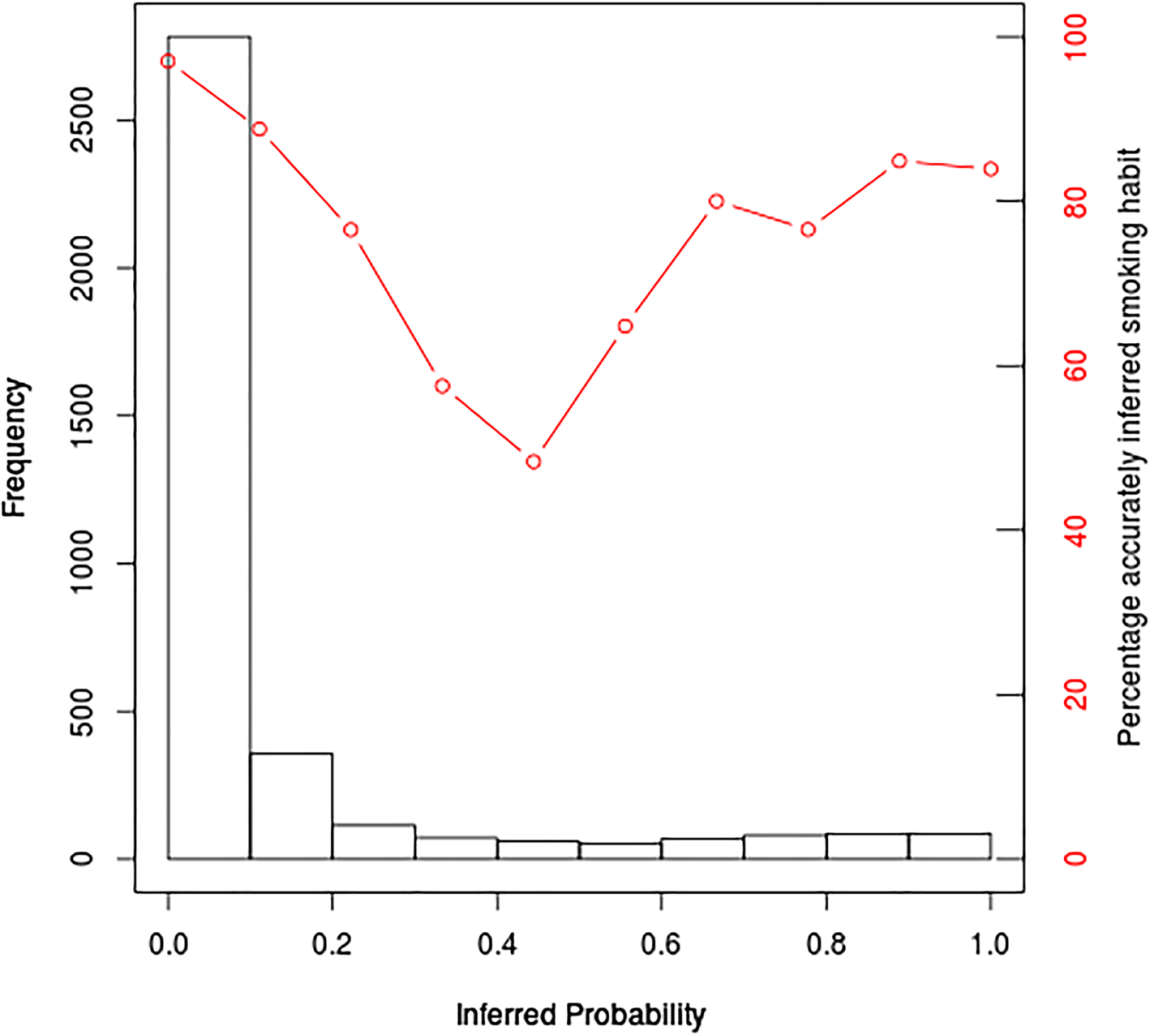
**Inferred probability of being a smoker versus the percentage of correctly inferred smoking habits.** Histogram of predicted probabilities in our model building dataset (N = 3764), probabilities determined using the 13 CpGs included in the final prediction model. The y-axis presents the number of individuals for whom the predicted probability of being a smoker was within the given probability range (x-axis). The red dots present the percentage of individuals in each probability bin that were accurately inferred using a > 0.5 probability threshold for being a smoker

To identify the minimal number of CpGs required to achieve maximum prediction accuracy, we additionally used Chi squared tests. Applying this backward approach, the first significant difference between two models was noted when we compared the model with and without cg09935388 (Table 1; Fig. 2). The combined marker elimination approach resulted in a finite set of DNA methylation markers comprising 13 CpGs (Table 1; Fig. 2). The AUC for the identified 13-CpG model was 0.901 for distinguishing between smokers versus non-smokers (for other prediction accuracy measures, see Table 2). The remaining 7 CpGs raised the cumulative AUC only on the 4th decimal i.e. from 0.9010 to 0.9016 (Table 1; Fig. 2). Hence, this finite set of 13 CpGs was used for subsequent prediction modeling. Using the 13-CpG model, we inferred the smoking status of the participants included in our model building dataset; the inferred probabilities are presented in a histogram in Fig. 3, where each probability bin is overlaid with the percentage of accurately inferred smoking habits in that probability range.

**Table 2:** Outcomes of the two-category-model (smokers vs. non-smokers) for inferring smoking habits from blood based on CpGs

	13-CpG model			10-CpG model^a^		
	Model building data set (N = 3764)		External validation	Model building data set (N = 3764)		External validation
	Model building	Fivefold cross-validation	KORA (N = 1608)	Model building	Fivefold cross-validation	SHIP-Trend (N = 244)
Accuracy^b^ (95% CI) ± SD	0.923 (0.914, 0.931)	0.921 ± 0.008	0.926 (0.912, 0.938)	0.917 (0.908, 0.926)	0.917 ± 0.011	0.873 (0.825, 0.912)
Specificity	0.976	0.976 ± 0.005	0.983	0.975	0.975 ± 0.006	0.995
Sensitivity	0.585	0.577 ± 0.044	0.580	0.548	0.551 ± 0.042	0.412
AUC	0.901	0.897 ± 0.137	0.911	0.896	0.893 ± 0.012	0.888

Cross-validation analysis results are presented as mean ± standard deviation

*AUC* Area under the curve

^a^Three CpGs (cg06126421, cg22132788 and cg05951221) are not included in the EPIC methylation microarray dataset from SHIP-Trend, this model is included here to demonstrate a second external validation in SHIP next to KORA with the full 13-CpG model

^b^Proportion accurately inferred smoking habits, 95% confidence interval (CI)

Adjusting the prediction model for age resulted in a minor AUC increase from 0.901 to 0.907, adjusting for sex from 0.901 to 0.903 and including both age and sex in the model increased the AUC slightly from 0.901 to 0.911 (Online Resource 1: Table S4). Additionally, we tested the influence of cell counts on the model accuracy. In the subset of participants for which cell count measures were available (N = 3402), our 13-CpG model without cell counts achieved an AUC of 0.906. Including the cell count measurements for monocytes, granulocytes and lymphocytes in our 13-CpG model, the AUC was almost identical at 0.907 (Online Resource 1: Table S5). Since age, sex and cell counts only had a minor impact on the prediction accuracy, these three non-epigenetic factors were not considered in the final model used in the subsequent analyses.

Next, we considered former smokers as an additional, separate category in the prediction model building based on the finite set of 13 CpGs, resulting in a three-category prediction model. To this end, we considered a subset of 2939 participants for which the relevant smoking habit information was available. We obtained for the current smokers (N = 364) an AUC of 0.928, for the former smokers (N = 1332) 0.772, and for the never smokers (N = 1243) 0.835 (for other accuracy measures, see Table 3). Additionally, we calculated smoking cessation time for the former smokers (N = 1332), and used the 13-CpGs to infer smoking cessation for ≥ 5 years (N = 1160) versus < 5 years (N = 172), which resulted in an AUC of 0.793, for ≥ 10 versus < 10 years smoking cessation time (N = 1028 and N = 304, respectively) an AUC of 0.778 was obtained and for ≥ 15 versus < 15 years smoking cessation time (N = 887 and N = 445, respectively) an AUC of 0.779 was obtained (Table 4).

**Table 3:** Outcomes of the three-category-model (current smokers vs. former smokers vs. never smokers) for inferring smoking habits from blood based on CpGs

*Model building data set(N = 2939):model building 13-CpG model*	Never (N = 1243)	Former (N = 1332)	Current (N = 364)
Specificity	0.746	0.770	0.997
Sensitivity	0.780	0.652	0.668
AUC	0.835	0.772	0.928
*Fivefold cross*-*validation*			
Specificity	0.739 ± 0.017	0.766 ± 0.053	0.975 ± 0.008
Sensitivity	0.769 ± 0.060	0.643 ± 0.039	0.669 ± 0.056
AUC	0.830 ± 0.019	0.766 ± 0.023	0.925 ± 0.021

*External replication in KORA (N = 1608): 13-CpG model*	Never (N = 675)	Former (N = 707)	Current (N = 226)
Specificity	0.539	0.870	0.980
Sensitivity	0.916	0.392	0.615
AUC	0.781	0.699	0.914

*Model building data set *(N = 2939): model building 10-CpG model^a^	Never (N = 1243)	Former (N = 1332)	Current (N = 364)
Specificity	0.749	0.737	0.974
Sensitivity	0.751	0.648	0.626
AUC	0.825	0.753	0.922
*Fivefold cross*-*validation*			
Specificity	0.745 ± 0.013	0.735 ± 0.042	0.975 ± 0.010
Sensitivity	0.747 ± 0.050	0.645 ± 0.026	0.627 ± 0.025
AUC	0.823 ± 0.018	0.748 ± 0.023	0.919 ± 0.019

*External replication in SHIP-Trend (N* = *244):10-CpG model*^a^	Never (N = 101)	Former (N = 92)	Current (N = 51)
Specificity	0.490	0.822	0.990
Sensitivity	0.891	0.315	0.451
AUC	0.778	0.654	0.882

Cross-validation analysis results are presented as mean ± standard deviation

*AUC* Area under the Curve

^a^Three CpGs (cg06126421, cg22132788 and cg05951221) are not included in the EPIC methylation microarray dataset from SHIP-Trend

**Table 4:** Outcomes of the two-category models for inferring smoking history (years of cessation time) in former smokers from blood based on 13 CpGs

	Former < 5 year versus Former ≥ 5 year cessation time			Former < 10 year versus Former ≥ 10 year cessation time			Former < 15 year versus Former ≥ 15 year cessation time		
	Model building data set (N = 1332)		External validation	Model building data set (N = 1332)		External validation	Model building data set (N = 1332)		External validation
	Model building	Fivefold cross-validation	KORA (N = 652)	Model building	Fivefold cross-validation	KORA (N = 652)	Model building	Fivefold cross-validation	KORA (N = 652)
Accuracy^a^ (95% CI) ± SD	0.725 (0.700, 0.749)	0.715 ± 0.020	0.830 (0.799, 0.858)	0.730 (0.705, 0.753)	0.721 ± 0.029	0.799 (0.766, 0.829)	0.732 (0.707, 0.756)	0.718 ± 0.016	0.759
Specificity	0.715	0.691 ± 0.090	0.494	0.694	0.682 ± 0.063	0.471	0.663	0.644 ± 0.033	0.449
Sensitivity	0.727	0.718 ± 0.026	0.879	0.740	0.733 ± 0.026	0.900	0.767	0.756 ± 0.015	0.902
AUC	0.793	0.774 ± 0.024	0.760	0.778	0.766 ± 0.033	0.764	0.779	0.767 ± 0.020	0.754

Cross-validation analysis results are presented as mean ± standard deviation

*AUC* Area under the curve

^a^Proportion accurately inferred smoking habits, 95% confidence interval (CI)

Furthermore, for the current smokers (N = 364) we calculated the pack-years (see methods) and used the 13 CpG markers to infer pack-years for ≥ 15 pack-years (N = 210) versus < 15 pack-years (N = 154), which resulted in an AUC of 0.815. For ≥ 10 versus < 10 pack-years (N = 246 and N = 118, respectively) an AUC of 0.846 was obtained (Table 5).

**Table 5:** Outcomes of model applications to infer smoking history (pack-years) in current smokers (N = 364) from blood based on CpGs

	13-CpG model			10-CpG model^a^		
	Model Building (N = 364)	Fivefold Cross-validation	KORA F4 (N = 224)	Model Building (N = 364)	Fivefold Cross-validation	SHIP-Trend (N = 41)
*More or less than 10 pack-years*						
Accuracy (95% CI)^b^	0.824 (0.781, 0.862)	0.783 ± 0.05	0.813 (0.755, 0.861)	0.808 (0.76, 0.847)	0.770 ± 0.035	0.805 (0.651, 0.912)
Specificity	0.644	0.577 ± 0.131	0.343	0.602	0.548 ± 0.14	0.778
Sensitivity	0.911	0.882 ± 0.045	0.899	0.907	0.879 ± 0.046	0.813
AUC	0.846	0.800 ± 0.068	0.796	0.834	0.809 ± 0.039	0.837
*More or less than 15 pack-years*						
Accuracy (95% CI)^b^	0.733 (0.685, 0.778)	0.719 ± 0.093	0.786 (0.726, 0.838)	0.728 (0.679, 0.773)	0.709 ± 0.059	0.659 (0.494, 0.799)
Specificity	0.617	0.600 ± 0.204	0.455	0.597	0.575 ± 0.143	0.533
Sensitivity	0.819	0.805 ± 0.042	0.894	0.824	0.808 ± 0.035	0.731
AUC	0.815	0.767 ± 0.102	0.752	0.786	0.757 ± 0.077	0.779

Cross-validation analysis results are presented as mean ± standard deviation

Pack-years were calculated as the number of cigarettes smoked per day divided by 20, multiplied by the total years of smoking

^a^Three CpGs (cg06126421, cg22132788 and cg05951221) are not included in the EPIC methylation microarray dataset from SHIP-Trend

^b^Proportion accurately inferred smoking habits; 95% CI, confidence interval; AUC, Area under the Curve

Finally, we combined the pack-years in current smokers, smoking cessation in former smokers with the never smokers (N = 2939) into one model for life-time smoking information inferring. We obtained for the current smokers with ≥ 15 pack-years (N = 210) an AUC of 0.949, < 15 pack-years (N = 154) an AUC of 0.869, in former smokers with ≤ 10 years smoking cessation (N = 311) an AUC of 0.793, with > 10 years smoking cessation (N = 1021) an AUC of 0.739 and the never smokers (N = 1243) an AUC of 0.835 (Table 6). We obtained for the current smokers with ≥ 10 pack-years (N = 246) an AUC of 0.948, < 10 pack-years (N = 118) an AUC of 0.863, former smokers with ≤ 10 years smoking cessation (N = 311) an AUC of 0.794, with > 10 years smoking cessation (N = 1021) an AUC of 0.739, and the never smokers (N = 1243) an AUC of 0.835 (Table 6).

**Table 6:** Outcomes of the five-category-model for inferring smoking habits and smoking history from blood based on 13 CpGs

Never versus former > 10 years cessation time versus former ≤ 10 years cessation time versus < 15 pack-years versus ≥ 15 pack-years					
*Model building data set (N = 2939)*	Never (N = 1243)	F > 10 year (N = 1021)	F ≤ 10 year (N = 311)	< 15PY (N = 154)	≥ 15PY (N = 210)
Specificity	0.712	0.777	0.979	0.987	0.967
Sensitivity	0.817	0.554	0.206	0.299	0.724
AUC	0.835	0.739	0.793	0.869	0.949
*Fivefold cross*-*validation*					
Specificity	0.711 ± 0.022	0.775 ± 0.036	0.977 ± 0.009	0.984 ± 0.009	0.963 ± 0.014
Sensitivity	0.809 ± 0.047	0.545 ± 0.040	0.199 ± 0.042	0.274 ± 0.128	0.695 ± 0.064
AUC	0.832 ± 0.014	0.731 ± 0.026	0.779 ± 0.018	0.855 ± 0.046	0.947 ± 0.016

*External replication in KORA (N = 1551)*	Never (N = 675)	F > 10 year (N = 488)	F ≤ 10 year (N = 164)	< 15 PY (N = 55)	≥ 15PY (N = 169)
Specificity	0.534	0.830	0.994	0.994	0.979
Sensitivity	0.927	0.299	0.122	0.018	0.728
AUC	0.788	0.650	0.791	0.710	0.955

Never versus former > 10 years cessation versus former ≤ 10 years cessation versus < 10 pack-years versus ≥ 10 pack-years					
*Model building data set (N = 2939)*	Never (N = 1243)	F > 10 year (N = 1021)	F ≤ 10 year (N = 311)	< 10 PY (N = 118)	≥ 10PY (N = 246)
Specificity	0.714	0.776	0.981	0.994	0.963
Sensitivity	0.817	0.554	0.193	0.220	0.772
AUC	0.835	0.739	0.794	0.863	0.948
*Fivefold cross*-*validation*					
Specificity	0.709 ± 0.023	0.774 ± 0.034	0.980 ± 0.006	0.992 ± 0.003	0.960 ± 0.008
Sensitivity	0.808 ± 0.045	0.542 ± 0.042	0.194 ± 0.043	0.206 ± 0.066	0.758 ± 0.067
AUC	0.831 ± 0.014	0.730 ± 0.027	0.780 ± 0.018	0.847 ± 0.047	0.946 ± 0.023

*External replication in KORA (N = 1551)*	Never (N = 675)	F > 10 year (N = 488)	F ≤ 10 year (N = 164)	< 10 PY (N = 35)	≥ 10PY (N = 189)
Specificity	0.535	0.827	0.994	0.998	0.977
Sensitivity	0.926	0.299	0.110	0.000	0.683
AUC	0.788	0.651	0.791	0.694	0.943

Cross-validation analysis results are presented as mean ± standard deviation

*AUC* area under the curve, *F* former smokers in years cessation time, *PY* pack-years

### Validating CpG-based models for inferring smoking habit and history from blood

We validated the newly developed prediction models based on the 13 selected CpGs via both internal and external validation procedures. Internal validation was carried out in the model building set using fivefold cross-validation and bootstrapping. For the two-category model (smokers vs. non-smokers), the optimism from bootstrap internal validation was 0.0032, resulting in a bootstrap-adjusted AUC of 0.898 (0.901–0.0032), see Table 2 for other accuracy measures and cross-validation results. For the three-category model (smokers vs. former smokers vs. never smokers) the bootstrap conducted optimisms are 0.0032 for current smokers, 0.0063 for former smokers and 0.0036 for never smokers resulting in bootstrap adjusted AUCs of 0.925 (0.928–0.0032) for current smokers, 0.766 (0.772–0.0063) for former smokers and 0.831 (0.835–0.0036) for never smokers (Table 3). For the smoking cessation time inference in former smoker, (1) for ≥ 5 versus < 5 years smoking cessation the bootstrap optimism was 0.0170 resulting in a bootstrap-adjusted AUC of 0.776 (0.793–0.0170); (2) for ≥ 10 versus < 10 years smoking cessation the bootstrap resulted in an optimism of 0.0112, giving a bootstrap-adjusted AUC of 0.767 (0.778–0.0112); (3) ≥ 15 versus < 15 years smoking cessation the bootstrap resulted in an optimism of 0.0096, giving a bootstrap-adjusted AUC of 0.769 (0.779–0.0096) (Table 4). For the two pack-year models, (1) the bootstrap optimism for ≥ 15 versus < 15 pack—was 0.029 resulting in a bootstrap-adjusted AUC of 0.786 (0.815–0.029); and (2) for ≥ 10 versus < 10 pack-years the bootstrap resulted in an optimism of 0.026, giving a bootstrap-adjusted AUC of 0.820 (0.846–0.026) (Table 5). Finally, for the life-time smoking information inferring, we obtained for ≥ 15 pack-years a bootstrap optimism of 0.0034 resulting in a bootstrap-adjusted AUC of 0.946 (0.949–0.0034), for < 15 pack-years a bootstrap-adjusted AUC of 0.860 (0.869–0.0091), for ≤ 10 smoking cessation a bootstrap-adjusted AUC of 0.782 (0.793–0.0106), > 10 years smoking cessation a bootstrap optimism of 0.0075 resulting in a bootstrap-adjusted AUC of 0.732 (0.739–0.0075) and for never smokers a bootstrap-adjusted AUC of 0.831 (0.835–0.0037) (Table 6). For the second five-category model, very similar results were obtained (Table 6).

External validation was performed in independent samples of two population-based studies, KORA and SHIP-Trend. In KORA (F4, N = 1608), an AUC of 0.911 was achieved for the full 13-CpG two-category model (Table 2). In SHIP-Trend (N = 244), an AUC of 0.888 was obtained for the two-category model based on a subset of ten CpGs, since the EPIC-array applied for SHIP-Trend is missing three of the 13 CpGs (cg06126421, cg22132788 and cg05951221). This 10-CpG model in the model building set gave a cross-validated average AUC of 0.893 ± 0.012 (Table 2). External validation of the three-category model in the KORA study (F4, N = 1608) achieved an AUC of 0.914 for the current smokers (N = 226), 0.699 for the former smokers (N = 707), and 0.781 for the never smokers (N = 675) (Table 3). The three-category model validation in SHIP-Trend for the 10-CpG model resulted in an AUC of 0.882 for current smokers (N = 51), 0.654 for former smokers (N = 92), and 0.778 for never smokers (N = 101) (Table 3). For comparison, in the model building set, this three category 10-CpG model gave a cross-validated average AUC of 0.919 ± 0.019 for current smokers, 0.748 ± 0.023 for former smokers, and 0.823 ± 0.018 for never smokers (Table 3). External validation of smoking cessation time inference in former smokers in the KORA study (N = 652) resulted in an AUC of 0.760 for ≥ 5 versus < 5 years of smoking cessation time, an AUC of 0.764 for ≥ 10 versus < 10 years of smoking cessation time, and of 0.754 for ≥ 15 versus < 15 years of smoking cessation time (Table 4). Furthermore, we externally validated the prediction of pack-years in the current smokers of the KORA study (F4, N = 224) and obtained an AUC of 0.752 for inferring ≥ 15 versus < 15 pack-years and an AUC of 0.796 for ≥ 10 versus < 10 pack-years (Table 5). The pack-year validation in the current smokers of SHIP-Trend (N = 41) for the 10-CpG model resulted in an AUC of 0.779 for ≥ 15 versus < 15 pack-years (AUC of 0.757 ± 0.077 in the model building set) and an AUC of 0.837 for ≥ 10 versus < 10 pack-years (AUC of 0.809 ± 0.039 in the model building) (Table 5). The external validation of the five-category models in the KORA study resulted for the current smokers with ≥ 15 pack-years in an AUC of 0.955, for < 15 pack-years an AUC of 0.710, for ≤ 10 years smoking cessation an AUC of 0.791, > 10 years smoking cessation an AUC of 0.650 and for never smokers an AUC of 0.788. For the second five-category model, we obtained in the KORA study an AUC of 0.943 for ≥ 10 pack-years, of 0.694 for < 15 pack-years, an AUC of 0.791 for ≤ 10 years smoking cessation, of 0.651 ≥ 10 years smoking cessation and an AUC of 0.788 for never smokers (Table 6).

### Comparing CpG-based with cotinine-based inference of smoking habit

In a subset of 488 participants for which we had CpG, cotinine and smoking information available, we compared our validated CpG-based prediction model for current versus non-smokers with the use of a cotinine cut-off to determine current smoking, using smoking information from self-reported questionnaires as reference. Using our CpG-model, we accurately inferred 87 of the 140 smokers and 344 of the 348 non-smokers (sensitivity of 0.621 and specificity of 0.989) compared to 105 of the 140 smokers and 342 of the 348 non-smokers using the cotinine level cut-off of 50 ng/mL (sensitivity of 0.750 and specificity of 0.983). Out of the 87 accurately inferred smokers with our CpG model, 75 (86%) were also accurately selected as smokers based on cotinine, and out of the 105 participants correctly selected with cotinine as smokers, 75 (71%) were accurately inferred as smokers with our CpG model. For the non-smokers, out of the 344 accurately inferred with our CpG model, 340 (99%) were also selected with cotinine as non-smokers, and 340 (99%) out of the 342 accurately selected non-smokers with cotinine, were accurately inferred as non-smokers with our CpG model. Finally, when comparing all three methods(questionnaires/cotinine levels/DNA methylation prediction), 340 participants were highlighted as non-smokers and 75 as smokers with all three methods, 12 were selected as smokers based on questionnaires and DNA methylation inference, 30 as smokers with both questionnaires and cotinine, 2 were determined as smokers with both cotinine and DNA methylation inference, whereas 23 were determined as smokers with questionnaires only, 2 as smokers with DNA methylation inference only, and 4 as smokers with cotinine only.

### Investigating prenatal smoking exposure effects on CpG-based inference of smoking habit

Next, we investigated the putative effect of prenatal smoking exposure and passive smoking on the epigenetic inference of smoking habits achievable with our validated model. When applying our model to the DNA methylation data at time of birth collected from cord blood, the proportion of children accurately inferred as non-smokers was surprisingly low at 0.114 (N = 1111) (Online Resource 1: Table S6). We then classified children whose mothers smoked throughout pregnancy as “smokers”, and obtained an AUC of 0.773, with a high sensitivity of 0.981 and a low specificity of 0.131. The AUC decreased to 0.664 when additionally considering mothers who stopped smoking when they became aware of their pregnancy (generally in the first trimester), and decreased further to 0.591 when additionally considering passive smoking of the mother during pregnancy; assessing the latter solely, an AUC of 0.460 was obtained, reflecting random prediction.

Additionally, we applied our model to data of children from the Generation R Study obtained from blood collected at the ages of six (N = 355) and ten (N = 309) years. In contrast to the results for newborns obtained from cord blood, we found that the proportion of 6- and 10-year-old children accurately inferred as non-smokers with our model was very high at 0.994 for both age groups (Table 7). This suggests no impact of prenatal smoking exposure nor passive smoking exposure during early childhood on the model performance. Subsequently, we applied our model to those 197 children for which epigenetic data were available from serial samples collected at birth, 6, and 10 years of age. The proportion of children that with our model accurately inferred as non-smokers at birth was 0.112, whereas it was 0.994 at six and 0.995 at 10 years of age, which was highly similar to the results obtained from the total datasets available for these three time points. The β-values per CpG for the model building set and the three time points in Generation R are shown in Online Resource 3: Figures S1–15.

**Table 7:** Model application to children from the Generation R study at 6 and 10 years of age

	Six years old	Six years old	Ten years old	Ten years old
	Whole dataset (N = 355)	Serial samples (N = 197)	Whole dataset (N = 309)	Serial samples (N = 197)
*Child non-smoking (all “0”)*				
Accuracy^a^	0.994	0.994	0.994	0.995
*Sustained prenatal smoking of mother throughout pregnancy*				
N	0:309	0:173	0:274	0:173
	1:46	1:24	1:35	1:24
Specificity	0.997	0.994	0.993	0.994
Sensitivity	0.022	0.0	0.0	0.0
AUC	0.649	0.650	0.606	0.592

*AUC* area under the curve

^a^Proportion of children correctly predicted as non-smokers

## Discussion

In this study, we introduce a robust, finite set of DNA methylation markers and carefully validated statistical models based on reasonably large population-based data, which together allow accurate and reliable inference of a person’s tobacco smoking habit and history from blood DNA.

Previous studies have identified numerous CpGs associated with tobacco smoking in blood, and showed that DNA methylation patterns of specific genes are modified by smoking habits [2, 21, 40–50]; here we took advantage of these EWASs as a marker discovery resource. From the 20 top smoking-associated CpGs consistently highlighted in previous EWASs and by using new population-based cohort data not overlapping with these previous EWASs, we identified a robust, finite set of 13 CpG markers as being most suitable for inferring a person’s smoking habit from blood DNA. Eight of these 13 CpGs are annotated to five known genes i.e., *AHRR* (2 CpGs), *GFI1* (2), *MYO1G* (2), *F2RL3* (1) and *PDZD2* (1), while the remaining 5 CpGs are not annotated to any coding regions. The highest AUC (0.880) for a given CpG among the 13 biomarkers in the model was achieved for cg05575921, which, together with one other CpG in the model (cg23576855), is located in the *AHRR* gene. The *AHRR* gene was shown to interact with the aryl hydrocarbon receptor (AHR), the induction point for the xenobiotic pathway, which includes several P450 enzymes, and is responsible for the degradation of environmental toxins [59–61]. Notably, *AHRR* provides the strongest epigenetic response to tobacco smoking known today [59, 62].

While a few previous studies have investigated DNA methylation markers for inferring smoking habits from blood, they all suffered from one or more limitations, including small sample size, limited model validation, exclusion of the former smoker category from the prediction model building, using a large number of CpGs and others [21–26]. For example, Philibert et al. [23] reported on the performance of five CpGs yielding AUCs 0.86–0.99 but only using 61 subjects. Notably, all five CpGs were among the 20 markers investigated in our study and are also included in our final 13-CpG model. For cg05575921, Philibert et al. estimated an AUC of 0.99 [23]; when testing this DNA methylation marker in our model building set of 3764 samples, a considerably lower AUC of 0.8801 was achieved. In another study, Elliot et al. [21] reported a methylation score based on 183 CpGs to distinguish between current, former and never smokers, with a sensitivity of 100% and a specificity of 97% using 96 subjects only. When generating the methylation score using the methods described by Elliot et al., and applying it to our model building set (N = 3764), we obtained a specificity of 0.864 and sensitivity of 0.747 with an AUC of 0.806, considerably lower than reported by Elliot et al. These two examples illustrate that previously reported prediction accuracies obtained from studies using small sample size likely reflect overestimation caused by small sample size. Given the relatively larger sample size for model building and internal validation, and for external validation with independent samples as utilized here, our results demonstrate that the new 13-CpG model introduced here provides more robust and reliable accuracy outcomes than previously reported models.

Previous studies have shown that DNA methylation patterns can be altered by age, sex and various lifestyle factors other than tobacco smoking [63, 64]. Additionally, recent papers suggest that the change in DNA methylation measurements due to smoking are mainly caused by the smoking induced changes in cell types [65–68]. We therefore tested the impact of age, sex and cell counts on the model performance and found that these covariates only provide a slight increase in the prediction accuracy our model provides. Notably, a model that does not consider sex, age and cell counts is beneficial for those applications where (some of) this information is not easily available, such as in forensics.

A recent study reported that the DNA methylation of most CpGs returns to never smoker levels within 5 years of smoking cessation, while some do not go back completely [11]. Also, previous work demonstrated that there is an association between smoking cessation time and smoking pack-years with DNA methylation scores [65, 69]. We therefore tested to what degree the 13 selected CpGs can distinguish former smokers from current smokers and never-smokers, and how well they allow inferring smoking history such as smoking cessation time and pack-years. Our results demonstrate that our 3-category model allows as first the inference of the former smoking category (smoking cessation between 0.1 and 58.86 years) together with current smokers and never smokers and also a more in depth inference possibility for cessation time categories as of more versus less than 5, 10 and 15 years of smoking cessation, although not as accurately as current and never smokers, as may be expected. The 13 CpGs also allowed accurate prediction of the pack-years in current smokers with a high AUCs for distinguishing between more or less than 10 pack-years, and for distinguishing between more or less than 15 pack-years. Finally, we show, to the best of our knowledge, for the first time an inference model able of inferring life-time smoking information in one model including the never smokers, cessation time in former smokers and pack-years in current smokers. Thus, the finite set of 13 DNA methylation markers and models we introduce here not only allow inferring information on current smoking or non-smoking status, but additionally provide information on former smoking and cessation time, smoking intensity in current smokers, and can additionally, as the first model to date, also provide complete life-time smoking information as of five different smoking categories.

Cotinine is the primary metabolite of nicotine and is therefore used as a reliable measurement for current smoking [19]. However, due to the short half-live of cotinine (between 15 and 19 h), a false-negative prediction of current smoking can be easily obtained when there is a long time between the last cigarette and blood drawn [19]. In addition, former smokers that use nicotine replacement therapy to reduce the motivation to smoke and for nicotine withdrawal symptoms, might result in false-positive predictions since cotinine, nicotine’s metabolite, will still be traceable [20, 70]. Finally, due to protein instability over time, cotinine levels would only be accurately measurable in fresh blood samples, which are not always available such as in forensic investigations. Zhang et al. [24] showed that both DNA methylation and cotinine can accurately distinguish current from never smokers, but also emphasized that only DNA methylation is able to provide more in depth life-time smoking information. In line with this, we show in the current study that using both cotinine (sensitivity 0.750, specificity 0.983) and DNA methylation (sensitivity 0.621, specificity 0.989) we can infer current smokers with high accuracy. However, the sensitivity of our CpG model is slightly lower than the use of the cotinine cut-off in this subset. Nonetheless, with the upcoming availability of DNA methylation data in large cohort studies, the availability of a reliable smoking inference model, giving extending life-time smoking information inference, would be more widely accessible than information on cotinine levels.

Maternal smoking during pregnancy has been shown to influence fetal DNA methylation patterns [57, 71], which in principle could affect epigenetic inference of smoking habits in adults. Additionally, it is shown that maternal smoking status can be predicted from DNA methylation retrieved from newborns [72, 73]. Therefore, we employed data from the Generation R study to test the influence of prenatal smoking exposure on the inference of smoking status in adolescence. Hence, we tested our prediction model using epigenetic data from cord blood collected at time of birth, and peripheral blood collected at 6 and 10 years of age [37]. Our results showed that at the age of 6 years, 353 of the 355 children were correctly inferred as non-smokers (accuracy of 0.994), and at the age of 10 years 307 of the 309 children (accuracy of 0.994) were correctly inferred as non-smokers. This might indicate that prenatal smoking exposure and passive smoking exposure does not affect DNA methylation levels to such an extent that they are detected with our inference model. At time of birth, our model incorrectly inferred 984 (88.57%) of the 1111 children as smokers (accuracy of 0.114). To test whether the newborns were inferred wrongly as smokers due to prenatal smoking exposure, we further classified the newborns as smokers when their mothers smoked throughout pregnancy (N = 161). This resulted in a high AUC (0.773), with high sensitivity (0.981) but low specificity (0.131). Retrieving this low specificity while correcting for prenatal smoking exposure may indicate that the incorrect smoking inference of newborns achieved with our model can only in part be explained by smoking exposure during pregnancy. Other explanations may be developmental effects, and perhaps the tissue difference between whole blood and cord blood and therefore the difference in cell composition, given that the applied model was developed using whole blood [74]. Previous studies have shown specific changes in DNA methylation during early childhood that were explained by developmental effects [71, 75]. In any case, given that envisioned applications of epigenetic inference of smoking habit in medical and forensic practice, as well as in most epidemiological and public health research, are typically performed in adults, our findings in children of advanced age imply that our model will indeed deliver smoking habit information of the adult individual tested, independent of prenatal smoking exposure or other effects.

The main strengths of our study are (1) the use of robust DNA methylation markers highlighted in multiple epigenome-wide association studies, (2) the use of independent population-based studies for marker discovery, model building and external model validation, and (3) the employment of thousands of samples for model building and validation. We therefore expect that the high prediction accuracy (AUC of 0.911) obtained from the full 13-CpG model in the KORA samples used for external validation reflects a realistic characterization of the performance of our model. This is also supported in part by the SHIP-Trend outcomes (AUC of 0.888) of the partial 10-CpG model. As the Illumina 450 K array on which our marker selection was initially based is no longer available, the SHIP-Trend results using 10-CpG subset from the current Infinium MethylationEPIC BeadChip indicate that this sub-model would be applicable to new studies moving forward.

This study, however, does not come without limitations. Our model is based on smoking habit data retrieved from self-reported questionnaires, which are generally considered unreliable in terms of underestimating actual smoking levels [15]. Regarding the putative inaccuracy of self-reported smoking habits used here as phenotypes, we cannot know how error-prone these reports are. In particular, it is possible that specific groups of volunteers, for instance pregnant women such as those involved in the Generation R Study, are more reluctant to confide that they smoke [16]. However, we did not use the Generation R Study data for model building or validation purposes. Moreover, we included cotinine data to confirm the self-reported smoking habits for subset of participants (N = 488). Overall, we expect that smoking phenotype inaccuracy did not strongly impact the performance outcomes of our models. Lastly, all but one of the studies included in the model building and model validation are population-based studies, which therefore can include participants with various diseases. Though, due to the large sample sizes used for model building and validation, we expect that disease status does not strongly impact our model performance. Another limitation for the pack-year model is the formula used to calculate the pack-years. For this estimation, the number of cigarettes the participant currently smokes is used, which might have changed over the life span, and if so, this phenotypic variation is not considered. Additionally, the start-age is used to calculate the number of years someone smoked or has been smoking, which might be prone to recall bias especially for elderly people.

We envision that future works may provide targeted laboratory tools for analysing the 13 CpGs included in our final model in different types of blood samples and possible translation to different tissues, as is recently already shown to be promising for our top hit CpG (cg0557592) in saliva [76]. This would enhance the spectrum of practical applications of epigenetic smoking habit inference. Given the finite set of DNA methylation markers introduced here, it is impractical to apply genome-wide DNA methylation microarrays just for the purpose of analyzing 13 CpGs. Moreover, there can be blood samples where microarrays do not produce reliable DNA methylation data, such as when the amount of DNA is low and/or the DNA is degraded such as DNA obtained from crime scene traces [17]. Hence, the future development of a fast and cheap laboratory tool that allows the reliable targeted analysis of the 13 CpGs highlighted here by employing a technology that can handle low quality and/or quantity DNA would be valuable. Foreseeing the future development of such a lab tool, we only included CpGs with at least a β-value difference ≥ 10% in mean or median (depending on availability per EWAS) in at least one published EWAS, to ensure detectability of the DNA methylation differences with targeted analysis technologies currently available [77, 78]. We view the positive results on epigenetic inference of smoking habits from blood presented here as a promising starting point for inferring more lifestyle factors using DNA methylation markers within the concept of epigenetic fingerprinting [17]. This requires continuous progress in identifying candidate DNA methylation predictors of lifestyle factors via dedicated EWASs, the subsequent use of these biomarkers in prediction modeling and validation studies to generate reliable and accurate models such as that reported here for tobacco smoking, and the development of robust and sensitive lab tools that allow the successful analysis of the DNA samples of interest, including those of limited quality and quantity.

## Electronic supplementary material

Below is the link to the electronic supplementary material.

**Online Resource 1:** In the Online Resource 1 we included the supplementary Tables 1–6. In table S1 and S2, we show the population characteristics of the model building dataset and the Generation R dataset, respectively. In table S3, we show the replication of the 20 top CpGs in their independent association with smoking status and their *p* value in the full model; smoking ~ all CpGs using the model building dataset. In Table S4 we show the prediction results for the inclusion of age and sex to the prediction model. In table S5, we show the results for the inclusion of cell count in a subset (N = 3402) to our model. Finally, we show the application of our model to children included in the Generation R study at birth (Table S6). (DOCX 58 kb)

**Online Resource 2:** In the Online Resource 2 we included the supplementary methods. We show per participating study a broader description of the study and the microarray data acquisition and preprocessing of the DNA methylation data used in the current study. (DOCX 69 kb)

**Online Resource 3:** In the Online Resource 3 we included the supplementary Figs. 1–13. The figures show for the 13 CpGs that are included in our model, per CpG, the DNA methylation β -values in the model building dataset (N = 3764) and in the Generation R study (N = 197) per age category. (DOCX 2450 kb)
