# Supplementary material for: Validated inference of smoking habits from blood with a finite DNA methylation marker set
Source: Eur J Epidemiol. 2019 Sep 7;34(11):1055–74. doi: 10.1007/s10654-019-00555-w (PMC6861351; doi:10.1007/s10654-019-00555-w)
Supplement: Supplementary file 1 — Online Resource 1: In the Online Resource 1 we included the supplementary Tables 1–6. In table S1 and S2, we show the population characteristics of the model building dataset and the Generation R dataset, respectively. In table S3, we show the replication of the 20 top CpGs in their independent association with smoking status and their p value in the full model; smoking ~ all CpGs using the model building dataset. In Table S4 we show the prediction results for the inclusion of age and sex to the prediction model. In table S5, we show the results for the inclusion of cell count in a subset (N = 3402) to our model. Finally, we show the application of our model to children included in the Generation R study at birth (Table S6). (DOCX 58 kb) [file 10654_2019_555_MOESM1_ESM.docx]

**Validated inference of smoking habits from blood with a finite DNA methylation marker set**

Silvana C.E. Maas^1,2^, Athina Vidaki^2^, Rory Wilson^3,4^, Alexander Teumer^5,6^, Fan Liu^2,7,8^, Joyce B.J. van Meurs^1,9^, André G. Uitterlinden^1,9^, Dorret I. Boomsma^10^, Eco J.C. de Geus^10^, Gonneke Willemsen^10^, Jenny van Dongen^10^, Carla J.H. van der Kallen^11^, P. Eline Slagboom^12^, Marian Beekman^12^, Diana van Heemst^13^, Leonard H. van den Berg^14^, BIOS Consortium, Liesbeth Duijts^15^, Vincent W.V. Jaddoe^1,16,17^, Karl-Heinz Ladwig^4^, Sonja Kunze^3,4^, Annette Peters^3,4,18,19^, M. Arfan Ikram^9^, Hans J. Grabe^20^, Janine F. Felix^1,16,17^, Melanie Waldenberger^3,4,18^, Oscar H. Franco^1^, Mohsen Ghanbari^1,21,* ,#^, and Manfred Kayser^2,*,#^

^1^ Department of Epidemiology, Erasmus MC University Medical Center Rotterdam, Rotterdam, the Netherlands  ^2^ Department of Genetic Identification, Erasmus MC University Medical Center Rotterdam, Rotterdam, the Netherlands

^3^ Research Unit of Molecular Epidemiology, Helmholtz Zentrum München, German Research Center for Environmental Health, Neuherberg, Germany

^4^ Institute of Epidemiology, Helmholtz Zentrum München, German Research Center for Environmental Health, Neuherberg, Germany

^5^ Institute for Community Medicine, University Medicine Greifswald, Greifswald, Germany

^6^ DZHK (German Center for Cardiovascular Research), partner site Greifswald, Greifswald, Germany

^7^ Key Laboratory of Genomic and Precision Medicine, Beijing Institute of Genomics, Chinese Academy of Sciences, Beijing, P.R. China

^8^ University of Chinese Academy of Sciences, Beijing, P.R. China
^9^ Department of Internal Medicine, Erasmus MC University Medical Center Rotterdam, Rotterdam, the Netherlands

^10^ Netherlands Twin Register, Dept. Biological Psychology, Vrije Universiteit, Amsterdam, the Netherlands

^11^ Department of Internal Medicine, Maastricht University Medical Centre, Maastricht, the Netherlands; Cardiovascular Research Institute Maastricht (CARIM), Maastricht University, Maastricht, the Netherlands
^12^ Molecular Epidemiology, dept. Biomedical Data Sciences, Biomedical Data Sciences, Leiden University Medical Center, Leiden, the Netherlands

^13^ Gerontology and geriatrics, dept. Internal Medicine, Biomedical Data Sciences, Leiden University Medical Center, Leiden, the Netherlands

^14^ Department of Neurology, Brain Center Rudolf Magnus, University Medical Center Utrecht, Utrecht, the Netherlands

^15^ Division of Respiratory Medicine and Allergology and Division of Neonatology, Department of Pediatrics, Erasmus MC University Medical Center Rotterdam, Rotterdam, the Netherlands

^16^ The Generation R Study Group, Erasmus MC University Medical Center Rotterdam, Rotterdam, the Netherlands

^17^ Department of Pediatrics, Erasmus MC University Medical Center Rotterdam, Rotterdam, the Netherlands

^18^ German Center for Cardiovascular Research (DZHK), Partner Site Munich Heart Alliance, Munich, Germany

^19^ Institute for Medical Informatics, Biometrics and Epidemiology, Ludwig-Maximilians-Universität (LMU) Munich, Munich, Germany

^20^ Department of Psychiatry and Psychotherapy, University Medicine Greifswald, Greifswald, Germany

^21^ Department of Genetics, School of Medicine, Mashhad University of Medical Science, Mashhad, Iran

# These authors contributed equally to this work.

***Correspondence:**

Manfred Kayser (m.kayser[@erasmusmc.nl](mailto:c.c.w.klaver@erasmusmc.nl)) or

Mohsen Ghanbari (m.ghanbari@erasmusmc.nl)

**Supplementary data**

**Table S1.** Characteristics of the dataset used for model building, internal (N=3,764) and external validation (N=1,852)

**Table S2.** Characteristics of Generation R Study data used for investigating the effect of prenatal smoking exposure

**Table S3.** Association and prediction results of the top 20 CpGs in the model building dataset (N=3,764)

**Table S4.** Prediction results when including age and sex in the model

**Table S5.** Prediction results when including cell count in the model

**Table S6.** Model application to children from the Generation R study at time of birth using cord blood

**Table S1. Characteristics of the dataset used for model building, internal (N=3,764) and external validation (N=1,852)**

| **Study** |  | | **No. of individuals** | | **Females (%)** | | **Average age (SD)** |
| --- | --- | --- | --- | --- | --- | --- | --- |
| **Model building and internal validation dataset (Dutch Europeans)** | | | | | | | |
| *Two-category model* | | *Total* | | 3,764 | 2,148 (57.07) | 53.65 (15.45) | |
|  | | Smokers | | 511 | 304 (59.49) | 49.66 (15.36) | |
|  | | Non-smokers^♦^ | | 3,253 | 1,844 (56.69) | 54.27 (15.38) | |
| *Three-category model* | | *Total* | | 2,939 | 1,670 (56.82) | 55.76 (15.17) | |
|  | | Smokers | | 364 | 218 (59.89) | 52.53 (14.51) | |
|  | | Former smokers | | 1,332 | 646 (48.50) | 61.06 (10.75) | |
|  | | Never smokers | | 1,243 | 807 (64.92) | 51.02 (17.40) | |
| **External model validation dataset (German Europeans)** | | | | | | | |
| **KORA F4** | | *Total* | | 1,608 | 831 (51.68) | 60.93 (8.83) | |
| *Two-category model* | | | |  |  |  | |
|  | | Smokers | | 226 | 106 (46.9) | 57.02 (6.79) | |
|  | | Non-smokers^♦^ | | 1,382 | 725 (52.46) | 61.57 (8.96) | |
| *Three-category model* | | | |  |  |  | |
|  | | Smokers | | 226 | 106 (46.9) | 57.02 (6.79) | |
|  | | Former smokers | | 707 | 277 (39.18) | 61.02 (8.96) | |
|  | | Never smokers | | 675 | 448 (66.37) | 62.14 (8.93) | |
| **SHIP-Trend** | | *Total* | | 244 | 127 (52) | 51.3(13.8) | |
| *Two-category model* | | | |  |  |  | |
|  | | Smokers | | 51 | 29 (56.9) | 45.7 (11.8) | |
|  | | Non-smokers^♦^ | | 193 | 95 (50.8) | 52.8 (13.9) | |
| *Three-category model* | | | |  |  |  | |
|  | | Smokers | | 51 | 29 (56.9) | 45.7 (11.8) | |
|  | | Former smokers | | 92 | 31 (33.7) | 53.3 (13.3) | |
|  | | Never smokers | | 101 | 67 (66.3) | 52.3 (14.5) | |

^♦^Non- smokers: Former smokers and never smokers combined

*SD* standard deviation.
*SHIP –Trend* Study of health in Pomerania,
*KORA* (F4) Kooperative Gesundheitsforschung in der Region Augsburg F4 Study
Model building and validation set contains participant data from 6 cohorts; Cohort on Diabetes and Atherosclerosis Maastricht (*CODAM*), LifeLines (*LL*), Leiden Longevity Study (*LLS*), Netherlands Twin Register (*NTR)*, Prospective ALS Study Netherlands (*PAN*), Rotterdam Study (*RS*).

**Table S2. Characteristics of Generation R Study data used for investigating the effect of prenatal smoking exposure**

|  |  |  | **Maternal smoking during pregnancy** | | | **Paternal / other smoking during pregnancy** | | | | |
| --- | --- | --- | --- | --- | --- | --- | --- | --- | --- | --- |
| **Sample set** | **No. of individuals** | **Females (%)** | **Whole**  **pregnancy** | **Until first trimester** | **Never** | **Yes** | **No** | | ^♦^**Missing** | |
| Birth | 1,111 | 545 (49.1) | 161 | 106 | 844 | 465 | 598 | | 48 | |
| 6 years old | 355 | 185 (52.3) | 46 | 39 | 270 | 130 | 194 | | 31 | |
| 10 years old | 309 | 150 (48.5) | 35 | 37 | 237 | 112 | 170 | | 27 | |
| Serial samples^♦♦^ | 197 | 95  (48.2) | 24 | 26 | 147 | 73 | 119 | | 5 | |
| ^♦^ Participants without information on paternal / passive smoking exposure.  ^♦♦^ Serial samples of 197 children measured at all three time-points | | | | | | | |  | |  |

| **Table S3. Association and prediction results of the top 20 CpGs in the model building dataset (N=3,764)** | | | | |
| --- | --- | --- | --- | --- |
|  | **Independent association with smoking habits**^♦♦^ | | **Association in the full model with smoking habits**^♦♦♦^ | |
| **Marker** | **Coefficient** | **p-value** | **Coefficient** | **p-value** |
| cg05575921 ^♦^ | -17.443 | < 2.22e-16 | -18.270 | < 2.22e-16 |
| cg13039251 ^♦^ | 9.241 | < 2.00e-16 | 5.492 | 2.81e-05 |
| cg03636183 ^♦^ | -13.909 | < 2.22e-16 | 6.755 | 2.86e-05 |
| cg12803068 ^♦^ | 6.479 | < 2.00e-16 | -9.766 | 6.07e-10 |
| cg22132788 ^♦^ | 9.800 | < 2.22e-16 | 13.670 | 5.27e-10 |
| cg06126421 ^♦^ | -9.415 | < 2.22e-16 | 4.658 | 3.02e-05 |
| cg21566642 ^♦^ | -20.218 | < 2.22e-16 | -3.677 | 0.033 |
| cg23576855 ^♦^ | -4.390 | < 2.22e-16 | -1.598 | 0.001 |
| cg15693572 ^♦^ | 4.189 | < 2.22e-16 | 2.368 | 0.055 |
| cg05951221 ^♦^ | -13.816 | < 2.22e-16 | 4.751 | 0.012 |
| cg01940273 ^♦^ | -16.834 | < 2.22e-16 | -5.432 | 0.017 |
| cg12876356 ^♦^ | -4.741 | < 2.22e-16 | 5.444 | 0.015 |
| cg09935388 ^♦^ | -7.205 | < 2.22e-16 | -3.213 | 0.023 |
| cg19572487 | -10.216 | < 2.00e-16 | -0.666 | 0.518 |
| cg19859270 | -47.049 | < 2.00e-16 | -2.716 | 0.456 |
| cg18146737 | -4.602 | < 2.22e-16 | -0.980 | 0.606 |
| cg21161138 | -17.857 | < 2.22e-16 | -0.937 | 0.598 |
| cg23480021 | 4.741 | < 2.22e-16 | -0.362 | 0.874 |
| cg21188533 | 3.638 | < 2.22e-16 | 0.106 | 0.870 |
| cg03274391 | 4.715 | < 2.22e-16 | 0.140 | 0.916 |
| ^♦^ CpGs included in our final model ^♦♦^ The association between the selected CpG sites and smoking habits (smokers vs non- smokers) is tested in our dataset using binominal regression adjusted for age and sex (e.g. smoking ~ CpG_1_ + age + sex)  ^♦♦♦^ The statistical summary from the full model; testing the association between smoking habits (smokers vs non- smokers) and all 20 CpGs included in our model building procedure using binominal regression (smoking ~ CpG_1-20_) | | | | |

**Table S4. Prediction results when including age and sex in the model**

|  | **13 CpGs** | **13 CpGs + Age** | **13 CpGs + Sex** | **13 CpGs + Age + Sex** |
| --- | --- | --- | --- | --- |
| Accuracy^$^ | 0.923 | 0.925 | 0.925 | 0.923 |
| (95% CI) | (0.914, 0.931) | (0.916, 0.933) | (0.916, 0.933) | (0.915, 0.932) |
| Specificity | 0.976 | 0.975 | 0.976 | 0.976 |
| Sensitivity | 0.585 | 0.603 | 0.595 | 0.589 |
| AUC | 0.901 | 0.907 | 0.903 | 0.911 |

^$^ Proportion accurately inferred smoking habits

95% CI: confidence interval; AUC: Area under the Curve.

**Table S5. Prediction results when including cell count in the model**

|  | **13 CpGs** | **13 CpGs + Cell count** |
| --- | --- | --- |
| Accuracy^$^ | 0.925 | 0.925 |
| (95% CI) | (0.915, 0.933) | (0.916, 0.934) |
| Specificity | 0.975 | 0.975 |
| Sensitivity | 0.616 | 0.618 |
| AUC | 0.906 | 0.907 |
| The table shows prediction results by including cell count in the model in 3,402 participants (477 current smokers and 2,925 non- smokers).  ^$^ Proportion accurately inferred smoking habits  95%CI: confidence interval; AUC: Area under the Curve. | | |

| Table S6. Model application to children from the Generation R study at time of birth using cord blood | | | |
| --- | --- | --- | --- |
|  |  | **Birth (N=1,111)** | **Birth (N=197)** |
|  |  | **Whole dataset** | **Serial samples** |
| Child non-smoking (all “0”) | Accuracy^$^ | 0.114 | 0.112 |
| Sustained prenatal smoking of mother throughout pregnancy | N | 0: 950 1: 161 | 0: 173 1: 24 |
|  | Specificity | 0.131 | 0.121 |
|  | Sensitivity | 0.981 | 0.958 |
|  | AUC | 0.773 | 0.751 |
| Sustained prenatal smoking of mother throughout pregnancy *or*  mother stopped smoking  when aware of pregnancy | N | 0: 844 1: 267 | 0: 147 1: 50 |
|  | Specificity | 0.133 | 0.129 |
|  | Sensitivity | 0.944 | 0.940 |
|  | AUC | 0.664 | 0.571 |
| Active *or* passive smoking of mother during pregnancy^♦^ | N | 0: 576 1: 535 | 0: 108 1: 89 |
|  | Specificity | 0.135 | 0.148 |
|  | Sensitivity | 0.908 | 0.932 |
|  | AUC | 0.591 | 0.562 |
| Only passive smoking of mother during pregnancy^♦♦^ | N | 0: 843 1: 268 | 0: 158 1: 39 |
|  | Specificity | 0.110 | 0.120 |
|  | Sensitivity | 0.873 | 0.923 |
|  | AUC | 0.460 | 0.512 |
| ♦ Active smoking: sustained smoking of mother throughout pregnancy or until mother became aware of pregnancy, generally in the first trimester, passive smoking: smoking of others in the pregnant mother’s household or at her place of work  ♦♦ Passive smoking: mother never smoked during pregnancy but others smoked in the pregnant mother’s household or at her place of work  ^$^ Proportion of children correctly predicted as non-smokers AUC: Area under the Curve | | | |
