# Supplementary material for: Validated inference of smoking habits from blood with a finite DNA methylation marker set
Source: Eur J Epidemiol. 2019 Sep 7;34(11):1055–74. doi: 10.1007/s10654-019-00555-w (PMC6861351; doi:10.1007/s10654-019-00555-w)
Supplement: Supplementary file 2 — Online Resource 2: In the Online Resource 2 we included the supplementary methods. We show per participating study a broader description of the study and the microarray data acquisition and preprocessing of the DNA methylation data used in the current study. (DOCX 69 kb) [file 10654_2019_555_MOESM2_ESM.docx]

**Validated inference of smoking habits from blood with a finite DNA methylation marker set**

Silvana C.E. Maas^1,2^, Athina Vidaki^2^, Rory Wilson^3,4^, Alexander Teumer^5,6^, Fan Liu^2,7,8^, Joyce B.J. van Meurs^1,9^, André G. Uitterlinden^1,9^, Dorret I. Boomsma^10^, Eco J.C. de Geus^10^, Gonneke Willemsen^10^, Jenny van Dongen^10^, Carla J.H. van der Kallen^11^, P. Eline Slagboom^12^, Marian Beekman^12^, Diana van Heemst^13^, Leonard H. van den Berg^14^, BIOS Consortium, Liesbeth Duijts^15^, Vincent W.V. Jaddoe^1,16,17^, Karl-Heinz Ladwig^4^, Sonja Kunze^3,4^, Annette Peters^3,4,18,19^, M. Arfan Ikram^9^, Hans J. Grabe^20^, Janine F. Felix^1,16,17^, Melanie Waldenberger^3,4,18^, Oscar H. Franco^1^, Mohsen Ghanbari^1,21,* ,#^, and Manfred Kayser^2,*,#^

^1^ Department of Epidemiology, Erasmus MC University Medical Center Rotterdam, Rotterdam, the Netherlands  ^2^ Department of Genetic Identification, Erasmus MC University Medical Center Rotterdam, Rotterdam, the Netherlands

^3^ Research Unit of Molecular Epidemiology, Helmholtz Zentrum München, German Research Center for Environmental Health, Neuherberg, Germany

^4^ Institute of Epidemiology, Helmholtz Zentrum München, German Research Center for Environmental Health, Neuherberg, Germany

^5^ Institute for Community Medicine, University Medicine Greifswald, Greifswald, Germany

^6^ DZHK (German Center for Cardiovascular Research), partner site Greifswald, Greifswald, Germany

^7^ Key Laboratory of Genomic and Precision Medicine, Beijing Institute of Genomics, Chinese Academy of Sciences, Beijing, P.R. China

^8^ University of Chinese Academy of Sciences, Beijing, P.R. China
^9^ Department of Internal Medicine, Erasmus MC University Medical Center Rotterdam, Rotterdam, the Netherlands

^10^ Netherlands Twin Register, Dept. Biological Psychology, Vrije Universiteit, Amsterdam, the Netherlands

^11^ Department of Internal Medicine, Maastricht University Medical Centre, Maastricht, the Netherlands; Cardiovascular Research Institute Maastricht (CARIM), Maastricht University, Maastricht, the Netherlands
^12^ Molecular Epidemiology, dept. Biomedical Data Sciences, Biomedical Data Sciences, Leiden University Medical Center, Leiden, the Netherlands

^13^ Gerontology and geriatrics, dept. Internal Medicine, Biomedical Data Sciences, Leiden University Medical Center, Leiden, the Netherlands

^14^ Department of Neurology, Brain Center Rudolf Magnus, University Medical Center Utrecht, Utrecht, the Netherlands

^15^ Division of Respiratory Medicine and Allergology and Division of Neonatology, Department of Pediatrics, Erasmus MC University Medical Center Rotterdam, Rotterdam, the Netherlands

^16^ The Generation R Study Group, Erasmus MC University Medical Center Rotterdam, Rotterdam, the Netherlands

^17^ Department of Pediatrics, Erasmus MC University Medical Center Rotterdam, Rotterdam, the Netherlands

^18^ German Center for Cardiovascular Research (DZHK), Partner Site Munich Heart Alliance, Munich, Germany

^19^ Institute for Medical Informatics, Biometrics and Epidemiology, Ludwig-Maximilians-Universität (LMU) Munich, Munich, Germany

^20^ Department of Psychiatry and Psychotherapy, University Medicine Greifswald, Greifswald, Germany

^21^ Department of Genetics, School of Medicine, Mashhad University of Medical Science, Mashhad, Iran

# These authors contributed equally to this work.

***Correspondence:**

Manfred Kayser (m.kayser[@erasmusmc.nl](mailto:c.c.w.klaver@erasmusmc.nl)) or

Mohsen Ghanbari ([m.ghanbari@erasmusmc.nl](mailto:m.ghanbari@erasmusmc.nl))

**Supplementary Methods**

***Study population characteristics***

This study was embedded within the Biobank-based Integrative Omics Study (BIOS) Consortium [1] which consists of six Dutch cohorts, namely the Rotterdam Study, Cohort on Diabetes and Atherosclerosis Maastricht, The Netherlands Twin Register, Leiden Longevity Study, Prospective ALS Study Netherlands, and LifeLines.

**The Rotterdam Study (RS)** [2] is a population-based cohort study in Rotterdam, the Netherlands. The design of the Rotterdam Study has been previously described in detail elsewhere [2]. In brief, the Rotterdam Study includes three sub-cohorts. In 1990, all residents of Ommoord, a district in Rotterdam, aged 45 years and older were invited to participate (RSI). In 2000, the cohort was extended to an additional 3,011 participants, who had reached the age 55 years, or who were 55 years and over and had moved into the research area (RSII). In 2006, a third cohort of 3,934 participants aged 45 years and older was initiated (RSIII). In our analysis, as part of the BIOS Consortium, we included 584 RS participants, of which 46 were smokers. We additionally included 646 independent RS participants not being part of the BIOS dataset, of which 116 were smokers.

**The Cohort on Diabetes and Atherosclerosis Maastricht (CODAM)** [3] consists of a selection of 547 subjects from a larger population-based cohort [4]. Inclusion of subjects into CODAM was based on a moderately increased risk of developing cardiometabolic diseases, such as type 2 diabetes and/or cardiovascular disease. Subjects were included if they were of European ancestry and over 40 years of age and additionally met at least one of the following criteria: increased body mass index (BMI; >25), a positive family history for type 2 diabetes, a history of gestational diabetes and/or glycosuria, or use of antihypertensive medication. We included 156 CODAM participants, of which 21 were smokers, in our analysis.

**The Netherlands Twin Register (NTR)** [5-7] was established in 1987 to study the extent to which genetic and environmental influences cause phenotypic differences between individuals. To this end, data from twins and their families (nearly 200,000 participants) from all over the Netherlands were collected, with a focus on health, lifestyle, personality, brain development, cognition, mental health and aging. We included 894 unrelated NTR participants, of which 152 were smokers, in our analysis.

**Leiden Longevity Study (LLS)** [8]. The aim of the LLS is to identify genetic factors influencing longevity and examine their interaction with the environment as a means to develop interventions to increase health at older ages. To this end, long-lived siblings of European descent were recruited together with their offspring and their offspring's partners, on the condition that at least two long-lived siblings were alive at the time of ascertainment. For men, the age criterion was 89 years or older; for women, the age criterion was 91 years or older. These criteria led to the ascertainment of 944 long-lived siblings from 421 families, together with 1,671 of their offspring and 744 partners. We included 625 LLS participants, of which 77 were smokers, in our analysis.

**Prospective ALS Study Netherlands (PAN)** [9] is a population-based study performed in the Netherlands. Patients diagnosed with suspected, possible, probable or definite ALS according to the El Escorial criteria and control samples were included. Individuals <15 years of age were excluded to avoid misclassification with juvenile onset of motor neuron diseases. Incident cases were identified from 1 January 2006 to 31 December 2009. Prevalent cases were all cases diagnosed before 31 December 2008 and still alive at that date. In 2016 there were more than 3,200 participants included in this study. We included 167 PAN participants, of which 8 were smokers, in our analysis.

**LifeLines- DEEP (LL)** [10] cohort is a sub-cohort of the LifeLines cohort [11]. LifeLines is a multidisciplinary prospective population-based cohort study examining the health and health-related behaviours of 167,729 individuals living in the northern parts of the Netherlands using a unique three-generation design. It employs a broad range of investigative procedures assessing biomedical, sociodemographic, behavioural, physical and psychological factors contributing to health and disease in the general population. A subset of 1,500 LifeLines participants also took part in LLD. For these participants, additional molecular data are generated, allowing for a more thorough investigation of the association between genetic and phenotypic variation. We included 692 LLD participants, of which 91 were smokers, in our analysis.

**Kooperative Gesundheitsforschung in der Region Augsburg (KORA) study** [12] consists of a series of independent population-based epidemiological surveys of participants living in the region of Augsburg, Southern Germany. All survey participants are residents, and of German nationality as identified through the registration office. The presented data were derived from the fourth population-based Cooperative Health Research in the Region of Augsburg (KORA) survey, S4. This cross-sectional survey covering the city of Augsburg (Germany) and two adjacent counties was conducted in 1999/2001, with 4,261 individuals aged 25 to 74 years. In a follow-up examination conducted in 2006/08 (KORA F4), 3,080 subjects participated. All participants underwent standardized examinations including blood withdrawals for plasma and DNA. A total of 1802 (F4) participants had methylation levels assessed. After excluding subjects failing quality control or lacking valid smoking or CpG data we included 1608 participants from the KORA F4 time point, 226 were smokers.

**The study of Health in Pomerania (SHIP)- Trend** [13] is a longitudinal population-based cohort study in West Pomerania, a region in the northeast of Germany, assessing the prevalence and incidence of common population-relevant diseases and their risk factors. Baseline examinations for SHIP-Trend were carried out between 2008 and 2012, comprising 4,420 participants aged 20 to 81 years. Study design and sampling methods were previously described [14]. The medical ethics committee of the University of Greifswald approved the study protocol, and oral and written informed consents were obtained from each of the study participants. We included 244 SHIP-Trend participants, of which 51 were smokers, in our analysis.

**The Generation R Study** [15] is a population-based prospective cohort study from fetal life onwards, conducted in Rotterdam. Pregnant women with an expected delivery date between April 2002 and January 2006 living in Rotterdam were eligible for participation in the study. Extensive assessments are performed in mothers, fathers and their children. The children form a prenatally recruited birth cohort that will be followed at least until young adulthood. Around the ages of 6 and 10 years all children and their parents were invited to visit the research center in the Erasmus MC-Sophia Children’s Hospital to participate in hands-on measurements, advanced imaging modalities, behavioral observations and biological sample collection. We included 1,111 children participating at birth, 355 at 6 years of age, and 309 at 9 years of age in our analyses, for whom information on DNA methylation and maternal smoking habits during pregnancy were available. All children were non-smokers, and 197 children overlapped between all three time points (at birth, 6 and 9 years old).

***Microarray data acquisition and preprocessing DNA methylation data***

**Cohorts included in the BIOS consortium [16]**The Zymo EZ DNA methylation kit (Zymo Research) was used to bisulfite-convert 500 ng of DNA per sample of which 4 µl was measured on the Illumina 450K array using the manufacturer’s protocol (Illumina Inc., San Diego, CA, USA.). The pipeline created by Tobi *et al.* [17] was used for the quality control and normalization of the generated data. In brief, MethylAid [18] was used to detect and remove outliers; hence, 95 samples were removed. Probes with a detection P value > 0.01, bead number < 3 or zero intensity were removed, as well as the ambiguously mapped probes. In total, there were 34,064 probes removed, resulting in a final dataset containing 453,109 CpGs [19]. Furthermore, the functional normalization [20] approach was implemented in minfi [21] (five principal components of the control probes) to normalize DNA methylation data; no samples or probes were removed (threshold of 5% probe failure or probes with 5% sample fail). The R code for the quality control and normalization pipeline is available at: <https://git.lumc.nl/molepi/Leiden450K>.

**Rotterdam Study**

DNA was extracted from whole peripheral blood (stored in EDTA tubes) by standardized salting out methods. 500 ng of DNA per sample was treated with sodium bisulfite using the Zymo EZ-96 DNA-methylation kit (Zymo Research). The samples were hybridized to the Illumina Human Methylation 450K array (Illumina Inc., San Diego, CA, USA.), according to the manufacturer’s protocol. Genome-wide DNA methylation levels were measured and methylation percentage per CpG marker was reported as a β-value ranging between 0 and 1 [22]. The data preprocessing was additionally performed using Genome Studio (v2011.1, methylation module version 1.9.0; Illumina). In total, 16 samples were removed: seven had a sample call rate below 99%, five had incomplete bisulfite conversion and four had sex mismatches. Probes with a calculated p-value by Genome Studio with a detection p value of more than 0.01 in more than 1% of the samples were excluded. Additionally, sample-level quality control was performed using MethylAid [18]. This resulted in a total set of 474,528 probes that were normalized using the Dasen option of the WateRmelon R-package [23]. Genome coordinates provided by Illumina (GRCh37/hg19) were used to identify independent loci.

**Kooperative Gesundheitsforschung in der Region Augsburg study (KORA)**

Genomic DNA (1 µg) was bisulfite-converted using the EZ-96 DNA Methylation Kit (Zymo Research) according to the manufacturer’s procedure, with the alternative incubation conditions recommended when using the Illumina Infinium Methylation Assay (Illumina Inc., San Diego, CA, USA.). Genome-wide DNA methylation was assessed using the Illumina HumanMethylation450 BeadChip, following the Illumina Infinium HD Methylation protocol. Data normalization from the data included in the KORA study was done following the CPACOR pipeline [24]. To summarize, 65 single-nucleotide polymorphism markers were excluded and background correction was done using the R package minfi [21]. Probes were set to N/A if the detection p-value ≥0.01 or number of beads ≤3. Samples were excluded if the detection rate was ≤0.95. Quantile normalization was then performed on the signal intensities separated into six different probe-type categories defined by colour channel, probe-type and M/U subtype (Type-I M red, Type-I U red, Type-I M green, Type-I U green, Type-II red, Type-II green). The percentage of methylation (β-value) of a given cytosine was calculated using these normalized intensities.

**The Study of Health in Pomerania (SHIP) - Trend**

DNA was extracted from blood samples of n=160 SHIP-Trend participants to assess DNA methylation using the Illumina HumanMethylationEPIC BeadChip array (Illumina Inc., San Diego, CA, USA.). Samples were randomly selected based on availability of multiple OMICS data taking the distribution of cardiovascular risk factors (hypertension, obesity, type II diabetes, smoking, and lipid levels) into account. The samples were taken between 07:00 AM and 04:00 PM, and serum aliquots were prepared for immediate analysis and for storage at -80 °C in the Integrated Research Biobank (Liconic, Liechtenstein). Processing of the DNA samples was performed at the Helmholtz Zentrum München. Preparation and normalization of the array data was performed according to the CPACOR workflow [24] using the software package R (www.r-project.org). The array idat files were processed using the minfi package. Probes that had a detection p-value above background (sum of per-array methylated and unmethylated intensity values based p-value ≥1E-16) were set to missing. Methylation beta values were calculated as proportion of methylated intensity value on the sum of methylated+unmethylated+100 intensities. Arrays with observed technical problems (±4SD outside control probe intensity mean) during steps like bisulfite conversion, hybridization or extension, as well as arrays with mismatch between sex of the proband and sex determined by the chr X and Y probe intensities were removed from subsequent analyses. Additionally, only arrays with a call rate ≥ 95% were processed further resulting in 248 samples with methylation data on 865,859 sites available for subsequent analyses.

**Generation R Study**

DNA extracted using the salting-out method from blood samples taken at birth (cord blood), at the 5-year or 9-year follow-up was used for this analysis. 500 ng of DNA per sample underwent bisulfite conversion using the EZ-96 DNA Methylation kit (Shallow) (Zymo Research Corporation). Samples were plated onto 96-well plates in no specific order. Samples were processed with the Illumina Infinium HumanMethylation450 BeadChip (Illumina Inc., San Diego, CA, USA.), which analyses methylation at 485,577 CpG sites. Preparation and normalization of the HumanMethylation450 BeadChip array data was performed according to the CPACOR workflow using the software package R. In detail, the idat files were read using the minfi package. Probes that had a detection p-value above background (based on sum of methylated and unmethylated intensity values) ≥ 1E-16 were set to missing per array. Next, the intensity values were stratified by autosomal and non-autosomal probes and quantile normalized for each of the six probe type categories separately: type II red/green, type I methylated red/green and type I unmethylated red/green. Beta values were calculated as proportion of methylated intensity value on the sum of methylated+unmethylated+100 intensities. Arrays with observed technical problems, such as failed bisulfite conversion, hybridization or extension, as well as arrays with a mismatch between sex of the proband and sex determined by the chr X and Y probe intensities were removed from subsequent analyses. Additionally, only arrays with a call rate > 95% per sample were processed further. The final dataset contained 1,339 samples at birth, 469 samples at age 6 and 425 samples at age 10. Methylation beta values outside the range of (25th percentile - 3*interquartile range, 75th percentile + 3*interquartile range) were set to missing.

***References***

1. Bonder MJ, Luijk R, Zhernakova DV, Moed M, Deelen P, Vermaat M, et al. Disease variants alter transcription factor levels and methylation of their binding sites. Nat Genet. 2017;49(1):131-8.

2. Ikram MA, Brusselle GGO, Murad SD, van Duijn CM, Franco OH, Goedegebure A, et al. The Rotterdam Study: 2018 update on objectives, design and main results. Eur J Epidemiol. 2017;32(9):807-50.

3. van Greevenbroek MM, Jacobs M, van der Kallen CJ, Vermeulen VM, Jansen EH, Schalkwijk CG, et al. The cross-sectional association between insulin resistance and circulating complement C3 is partly explained by plasma alanine aminotransferase, independent of central obesity and general inflammation (the CODAM study). Eur J Clin Invest. 2011;41(4):372-9.

4. van Dam RM, Boer JM, Feskens EJ, Seidell JC. Parental history of diabetes modifies the association between abdominal adiposity and hyperglycemia. Diabetes Care. 2001;24(8):1454-9.

5. Willemsen G, Vink JM, Abdellaoui A, den Braber A, van Beek JH, Draisma HH, et al. The Adult Netherlands Twin Register: twenty-five years of survey and biological data collection. Twin Res Hum Genet. 2013;16(1):271-81.

6. Boomsma DI, Vink JM, van Beijsterveldt TC, de Geus EJ, Beem AL, Mulder EJ, et al. Netherlands Twin Register: a focus on longitudinal research. Twin Res. 2002;5(5):401-6.

7. Boomsma DI, Willemsen G, Sullivan PF, Heutink P, Meijer P, Sondervan D, et al. Genome-wide association of major depression: description of samples for the GAIN Major Depressive Disorder Study: NTR and NESDA biobank projects. Eur J Hum Genet. 2008;16(3):335-42.

8. Schoenmaker M, de Craen AJ, de Meijer PH, Beekman M, Blauw GJ, Slagboom PE, et al. Evidence of genetic enrichment for exceptional survival using a family approach: the Leiden Longevity Study. Eur J Hum Genet. 2006;14(1):79-84.

9. Huisman MH, de Jong SW, van Doormaal PT, Weinreich SS, Schelhaas HJ, van der Kooi AJ, et al. Population based epidemiology of amyotrophic lateral sclerosis using capture-recapture methodology. J Neurol Neurosurg Psychiatry. 2011;82(10):1165-70.

10. Tigchelaar EF, Zhernakova A, Dekens JA, Hermes G, Baranska A, Mujagic Z, et al. Cohort profile: LifeLines DEEP, a prospective, general population cohort study in the northern Netherlands: study design and baseline characteristics. BMJ Open. 2015;5(8):e006772.

11. Scholtens S, Smidt N, Swertz MA, Bakker SJ, Dotinga A, Vonk JM, et al. Cohort Profile: LifeLines, a three-generation cohort study and biobank. Int J Epidemiol. 2015;44(4):1172-80.

12. Holle R, Happich M, Lowel H, Wichmann HE, Group MKS. KORA--a research platform for population based health research. Gesundheitswesen. 2005;67 Suppl 1:S19-25.

13. Jurgens C, Volzke H, Tost F. [Study of health in Pomerania (SHIP-Trend): : Important aspects for healthcare research in ophthalmology]

Study of Health in Pomerania (SHIP-Trend) : Wichtige Aspekte fur die ophthalmologische Versorgungsforschung. Ophthalmologe. 2014;111(5):443-7.

14. Volzke H, Alte D, Schmidt CO, Radke D, Lorbeer R, Friedrich N, et al. Cohort profile: the study of health in Pomerania. Int J Epidemiol. 2011;40(2):294-307.

15. Kooijman MN, Kruithof CJ, van Duijn CM, Duijts L, Franco OH, van IMH, et al. The Generation R Study: design and cohort update 2017. Eur J Epidemiol. 2016;31(12):1243-64.

16. Dekkers KF, van Iterson M, Slieker RC, Moed MH, Bonder MJ, van Galen M, et al. Blood lipids influence DNA methylation in circulating cells. Genome Biol. 2016;17(1):138.

17. Tobi EW, Slieker RC, Stein AD, Suchiman HE, Slagboom PE, van Zwet EW, et al. Early gestation as the critical time-window for changes in the prenatal environment to affect the adult human blood methylome. Int J Epidemiol. 2015;44(4):1211-23.

18. van Iterson M, Tobi EW, Slieker RC, den Hollander W, Luijk R, Slagboom PE, et al. MethylAid: visual and interactive quality control of large Illumina 450k datasets. Bioinformatics. 2014;30(23):3435-7.

19. Chen YA, Lemire M, Choufani S, Butcher DT, Grafodatskaya D, Zanke BW, et al. Discovery of cross-reactive probes and polymorphic CpGs in the Illumina Infinium HumanMethylation450 microarray. Epigenetics. 2013;8(2):203-9.

20. Fortin JP, Labbe A, Lemire M, Zanke BW, Hudson TJ, Fertig EJ, et al. Functional normalization of 450k methylation array data improves replication in large cancer studies. Genome Biol. 2014;15(12):503.

21. Aryee MJ, Jaffe AE, Corrada-Bravo H, Ladd-Acosta C, Feinberg AP, Hansen KD, et al. Minfi: a flexible and comprehensive Bioconductor package for the analysis of Infinium DNA methylation microarrays. Bioinformatics. 2014;30(10):1363-9.

22. Sandoval J, Heyn H, Moran S, Serra-Musach J, Pujana MA, Bibikova M, et al. Validation of a DNA methylation microarray for 450,000 CpG sites in the human genome. Epigenetics. 2011;6(6):692-702.

23. Pidsley R, CC YW, Volta M, Lunnon K, Mill J, Schalkwyk LC. A data-driven approach to preprocessing Illumina 450K methylation array data. BMC Genomics. 2013;14:293.

24. Lehne B, Drong AW, Loh M, Zhang W, Scott WR, Tan ST, et al. A coherent approach for analysis of the Illumina HumanMethylation450 BeadChip improves data quality and performance in epigenome-wide association studies. Genome Biol. 2015;16:37.
