# Supplementary material for: Validated inference of smoking habits from blood with a finite DNA methylation marker set
Source: Eur J Epidemiol. 2019 Sep 7;34(11):1055–74. doi: 10.1007/s10654-019-00555-w (PMC6861351; doi:10.1007/s10654-019-00555-w)
Supplement: Supplementary file 3 — Online Resource 3: In the Online Resource 3 we included the supplementary Figs. 1–13. The figures show for the 13 CpGs that are included in our model, per CpG, the DNA methylation β -values in the model building dataset (N = 3764) and in the Generation R study (N = 197) per age category. (DOCX 2450 kb) [file 10654_2019_555_MOESM3_ESM.docx]

**Validated inference of smoking habits from blood with a finite DNA methylation marker set**

Silvana C.E. Maas^1,2^, Athina Vidaki^2^, Rory Wilson^3,4^, Alexander Teumer^5,6^, Fan Liu^2,7,8^, Joyce B.J. van Meurs^1,9^, André G. Uitterlinden^1,9^, Dorret I. Boomsma^10^, Eco J.C. de Geus^10^, Gonneke Willemsen^10^, Jenny van Dongen^10^, Carla J.H. van der Kallen^11^, P. Eline Slagboom^12^, Marian Beekman^12^, Diana van Heemst^13^, Leonard H. van den Berg^14^, BIOS Consortium, Liesbeth Duijts^15^, Vincent W.V. Jaddoe^1,16,17^, Karl-Heinz Ladwig^4^, Sonja Kunze^3,4^, Annette Peters^3,4,18,19^, M. Arfan Ikram^9^, Hans J. Grabe^20^, Janine F. Felix^1,16,17^, Melanie Waldenberger^3,4,18^, Oscar H. Franco^1^, Mohsen Ghanbari^1,21,*,#^, and Manfred Kayser^2,*, #^

^1^ Department of Epidemiology, Erasmus MC University Medical Center Rotterdam, Rotterdam, the Netherlands  ^2^ Department of Genetic Identification, Erasmus MC University Medical Center Rotterdam, Rotterdam, the Netherlands

^3^ Research Unit of Molecular Epidemiology, Helmholtz Zentrum München, German Research Center for Environmental Health, Neuherberg, Germany

^4^ Institute of Epidemiology, Helmholtz Zentrum München, German Research Center for Environmental Health, Neuherberg, Germany

^5^ Institute for Community Medicine, University Medicine Greifswald, Greifswald, Germany

^6^ DZHK (German Center for Cardiovascular Research), partner site Greifswald, Greifswald, Germany

^7^ Key Laboratory of Genomic and Precision Medicine, Beijing Institute of Genomics, Chinese Academy of Sciences, Beijing, P.R. China

^8^ University of Chinese Academy of Sciences, Beijing, P.R. China
^9^ Department of Internal Medicine, Erasmus MC University Medical Center Rotterdam, Rotterdam, the Netherlands

^10^ Netherlands Twin Register, Dept. Biological Psychology, Vrije Universiteit, Amsterdam, the Netherlands

^11^ Department of Internal Medicine, Maastricht University Medical Centre, Maastricht, the Netherlands; Cardiovascular Research Institute Maastricht (CARIM), Maastricht University, Maastricht, the Netherlands
^12^ Molecular Epidemiology, dept. Biomedical Data Sciences, Biomedical Data Sciences, Leiden University Medical Center, Leiden, the Netherlands

^13^ Gerontology and geriatrics, dept. Internal Medicine, Biomedical Data Sciences, Leiden University Medical Center, Leiden, the Netherlands

^14^ Department of Neurology, Brain Center Rudolf Magnus, University Medical Center Utrecht, Utrecht, the Netherlands

^15^ Division of Respiratory Medicine and Allergology and Division of Neonatology, Department of Pediatrics, Erasmus MC University Medical Center Rotterdam, Rotterdam, the Netherlands

^16^ The Generation R Study Group, Erasmus MC University Medical Center Rotterdam, Rotterdam, the Netherlands

^17^ Department of Pediatrics, Erasmus MC University Medical Center Rotterdam, Rotterdam, the Netherlands

^18^ German Center for Cardiovascular Research (DZHK), Partner Site Munich Heart Alliance, Munich, Germany

^19^ Institute for Medical Informatics, Biometrics and Epidemiology, Ludwig-Maximilians-Universität (LMU) Munich, Munich, Germany

^20^ Department of Psychiatry and Psychotherapy, University Medicine Greifswald, Greifswald, Germany

^21^ Department of Genetics, School of Medicine, Mashhad University of Medical Science, Mashhad, Iran

# These authors contributed equally to this work.

***Correspondence:**

Manfred Kayser (m.kayser[@erasmusmc.nl](mailto:c.c.w.klaver@erasmusmc.nl)) or

Mohsen Ghanbari ([m.ghanbari@erasmusmc.nl](mailto:m.ghanbari@erasmusmc.nl))

**Supplementary Figures**

**Fig. S1** β-values of cg05575921 in the model building and the Generation R Study datasets

**Fig. S2** β-values of cg13039251 in the model building and the Generation R Study datasets

**Fig. S3** β-values of cg03636183 in the model building and the Generation R Study datasets

**Fig. S4** β-values of cg12803068 in the model building and the Generation R Study datasets
**Fig. S5** β-values of cg22132788 in the model building and the Generation R Study datasets
**Fig. S6** β-values of cg06126421 in the model building and the Generation R Study datasets
**Fig. S7** β-values of cg21566642 in the model building and the Generation R Study datasets
**Fig. S8** β-values of cg23576855 in the model building and the Generation R Study datasets
**Fig. S9** β-values of cg15693572 in the model building and the Generation R Study datasets
**Fig. S10** β-values of cg059521221in the model building and the Generation R Study datasets
**Fig. S11** β-values of cg01940273 in the model building and the Generation R Study datasets
**Fig. S12** β-values of cg12876356in the model building and the Generation R Study datasets
**Fig. S13** β-values of cg09935388 in the model building and the Generation R Study datasets

**Figure legend**:

**Fig. S1-S13** show β -values of the 13 CpGs in the model building (N= 3,764) and Generation R study (N=197). Smoking habit in the model building dataset is coded as non-smokers (“0”) and current smokers (“1”). Maternal smoking habit during pregnancy is coded as non-smokers during pregnancy (“1”), mothers that stopped smoking during the first trimester (“2”) and mothers that smoked throughout pregnancy (“3”). The median and standard deviation are also shown in the Figures.

| **Fig. S1** β-values of cg05575921 in the model building and the Generation R Study datasets |
| --- |
|  |
| 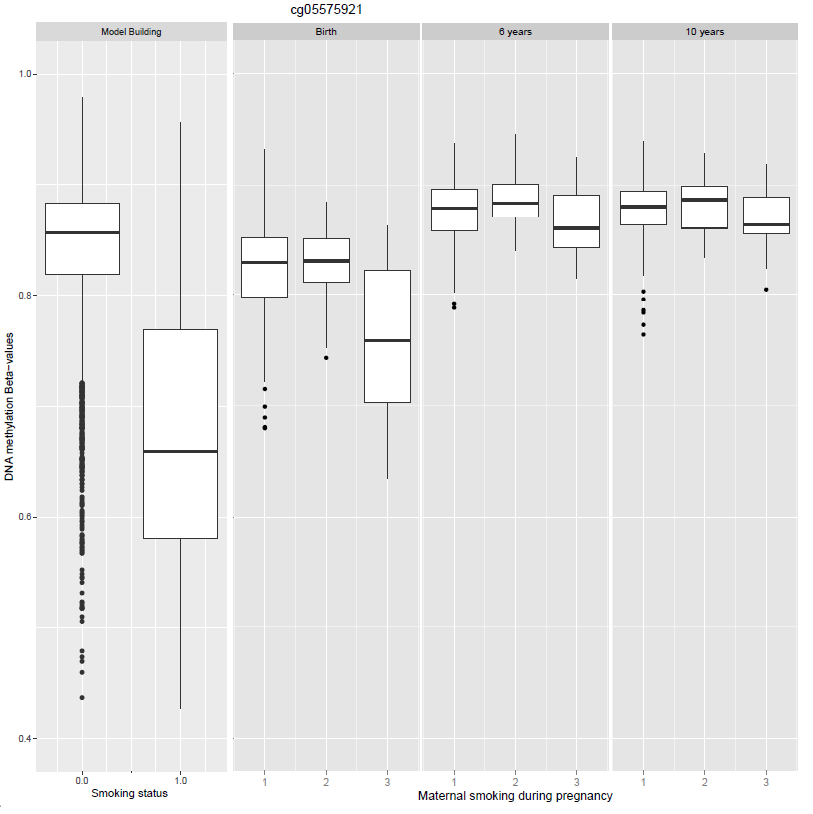 |

| **Fig. S2** β-values of cg13039251 in the model building and the Generation R Study datasets |
| --- |
| 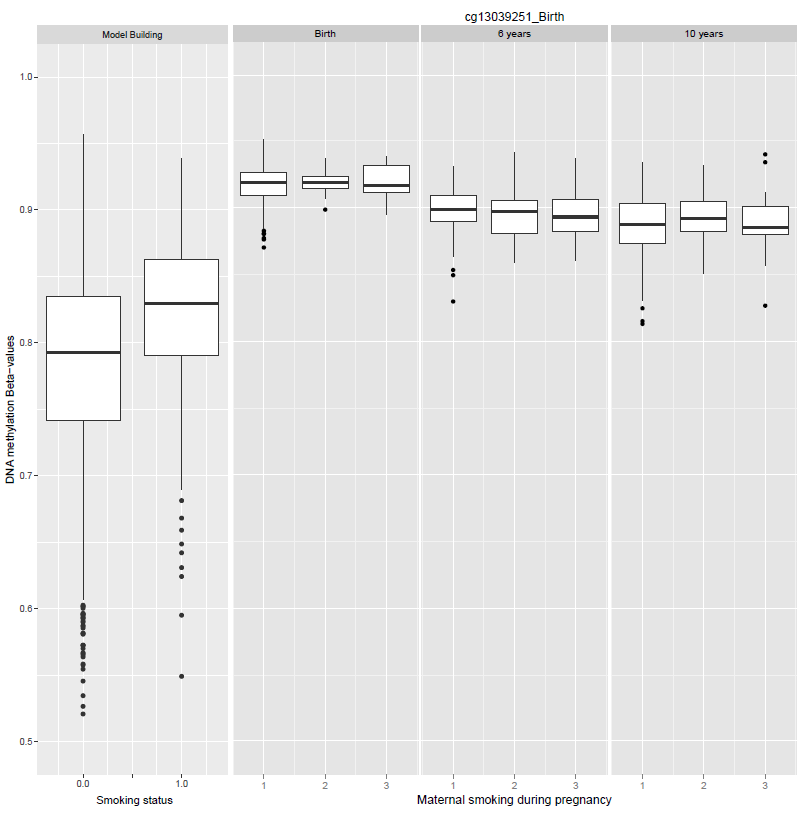 |
|  |

|  |
| --- |
|  |

| **Fig. S3** β-values of cg03636183 in the model building and the Generation R Study datasets |
| --- |
| 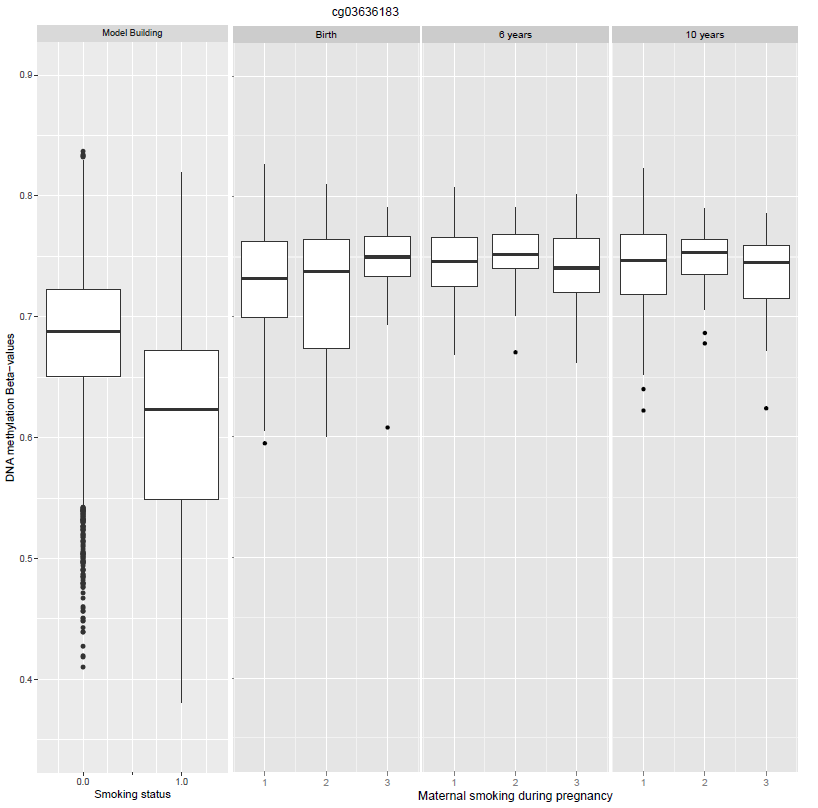 |

| **Fig. S4** β-values of cg12803068 in the model building and the Generation R Study datasets |
| --- |
| 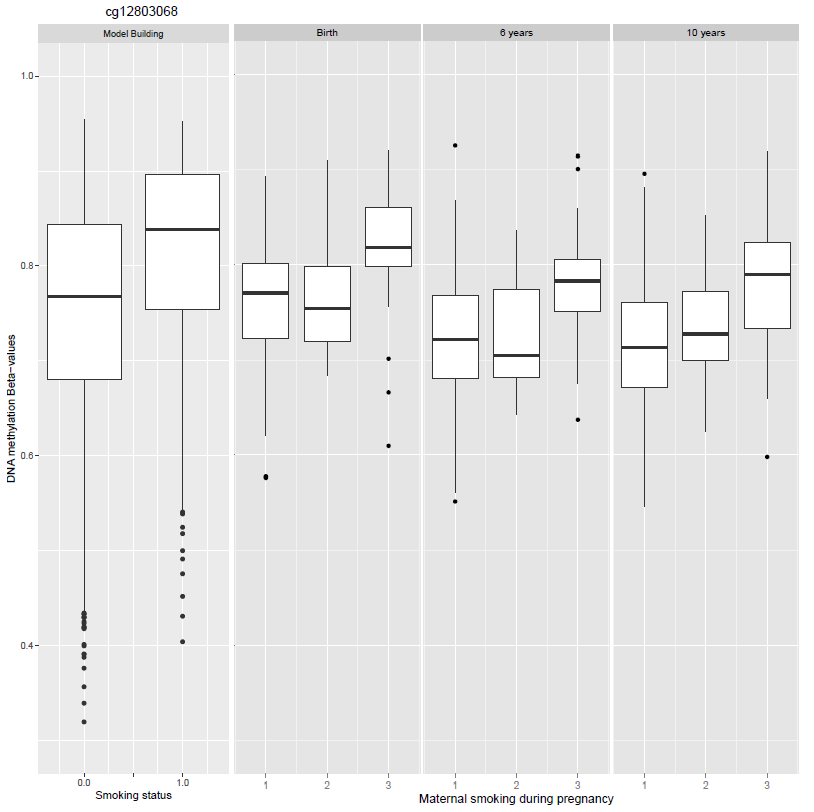 |

| **Fig. S5** β-values of cg22132788 in the model building and the Generation R Study datasets |
| --- |
|  |
| 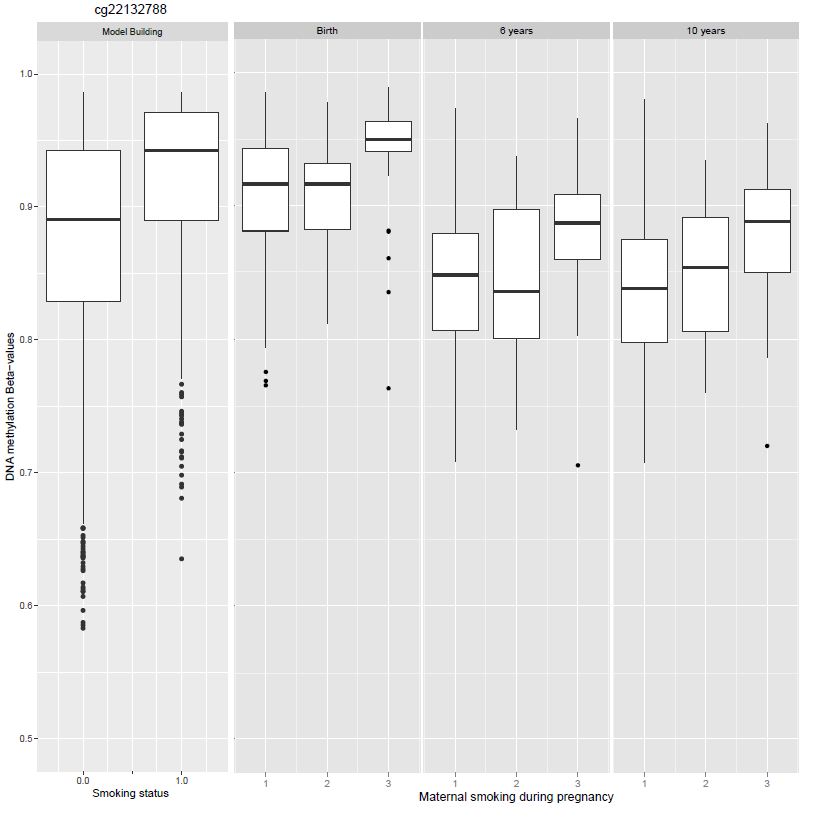 |

| **Fig. S6** β-values of cg06126421 in the model building and the Generation R Study datasets |
| --- |
| 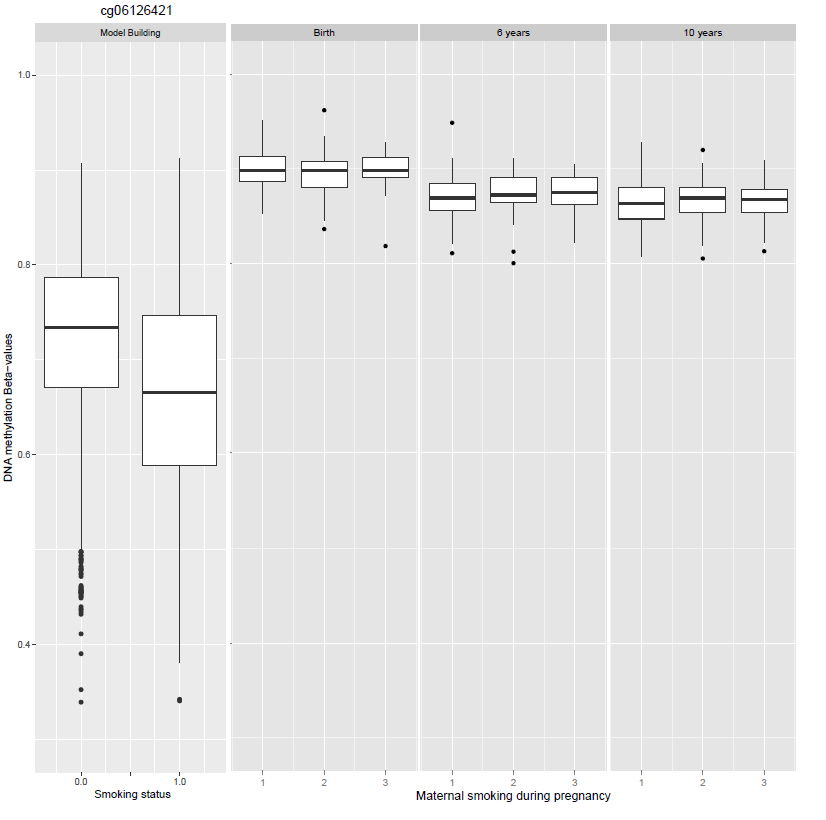 |
|  |

| **Fig. S7** β-values of cg21566642 in the model building and the Generation R Study datasets |
| --- |
| 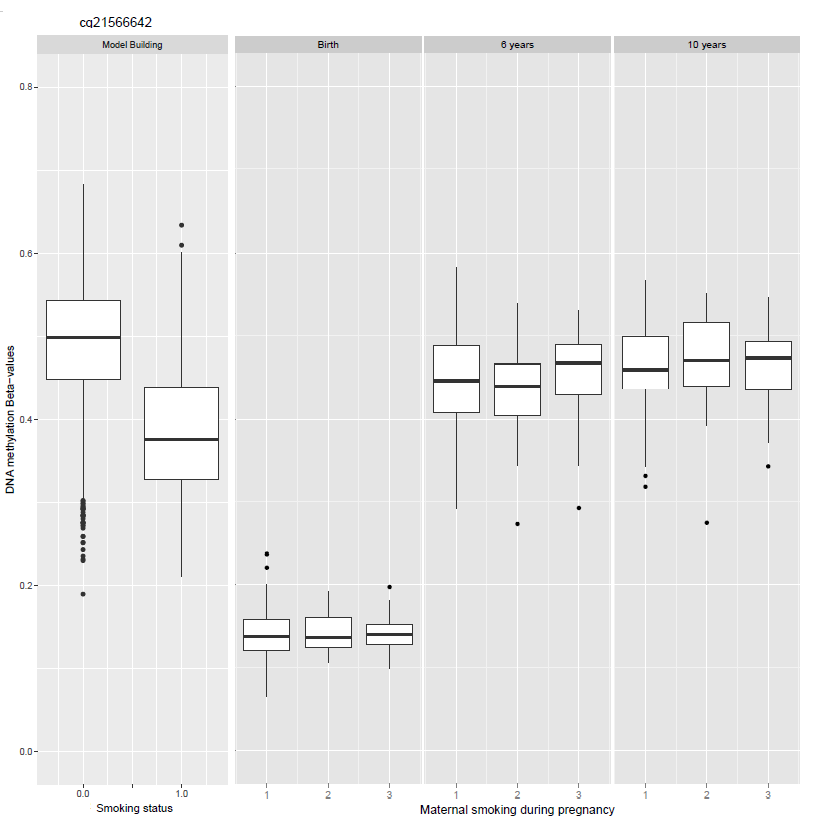 |
|  |

| **Fig. S8** β-values of cg23576855 in the model building and the Generation R Study datasets |
| --- |
| 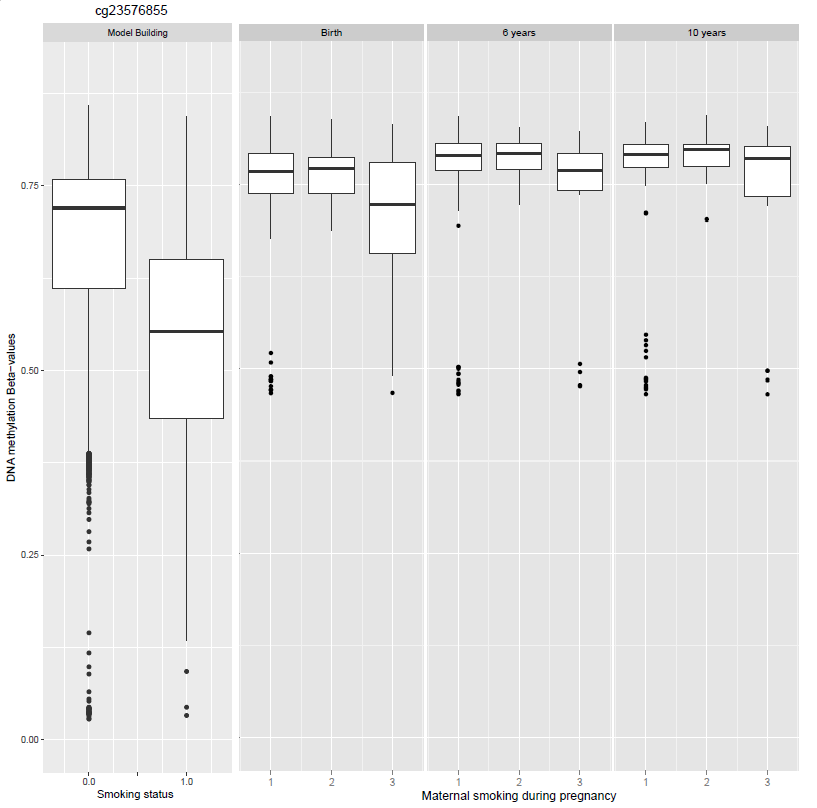 |
|  |

| **Fig. S9** β-values of cg15693572 in the model building and the Generation R Study datasets |
| --- |
| 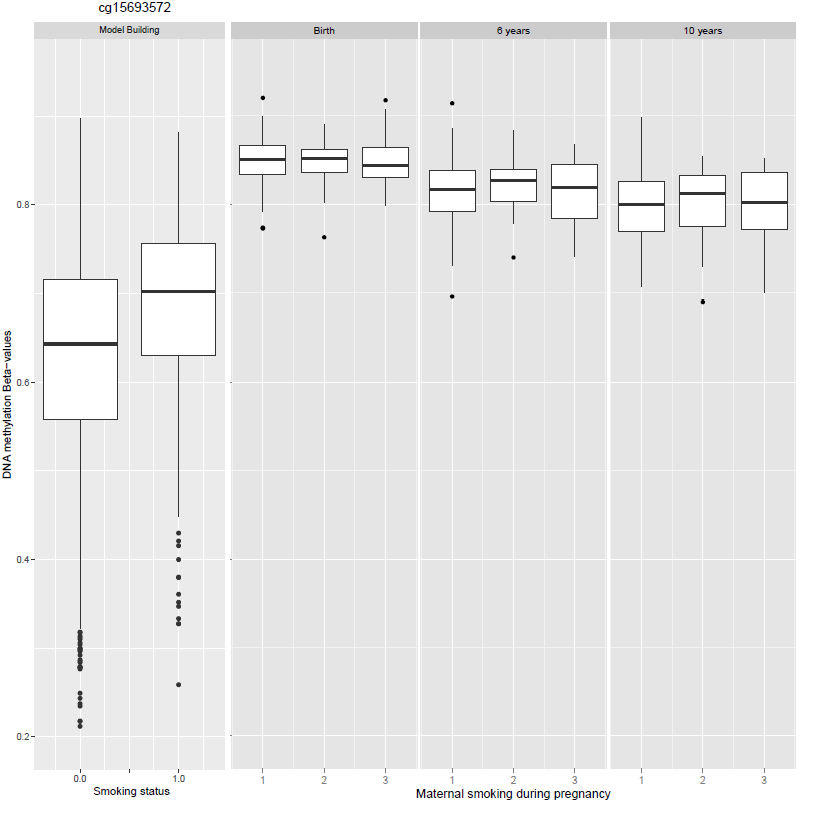 |
|  |

| **Fig. S10** β-values of cg05951221 in the model building and the Generation R Study datasets |
| --- |
| 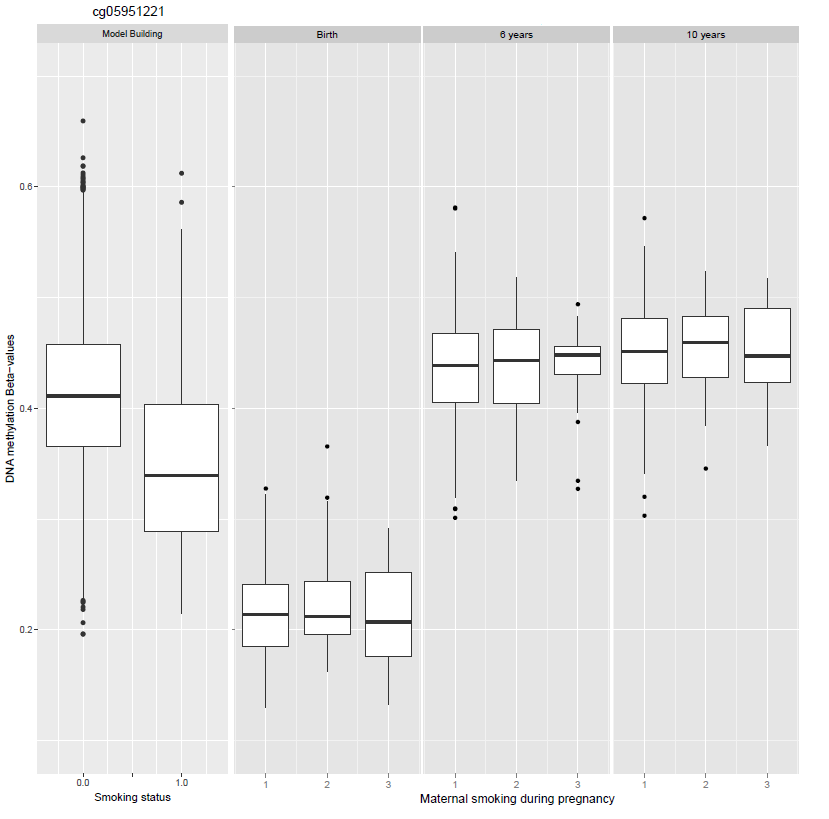 |
|  |

| **Fig. S11** β-values of cg01940273 in the model building and the Generation R Study datasets |
| --- |
|  |
| 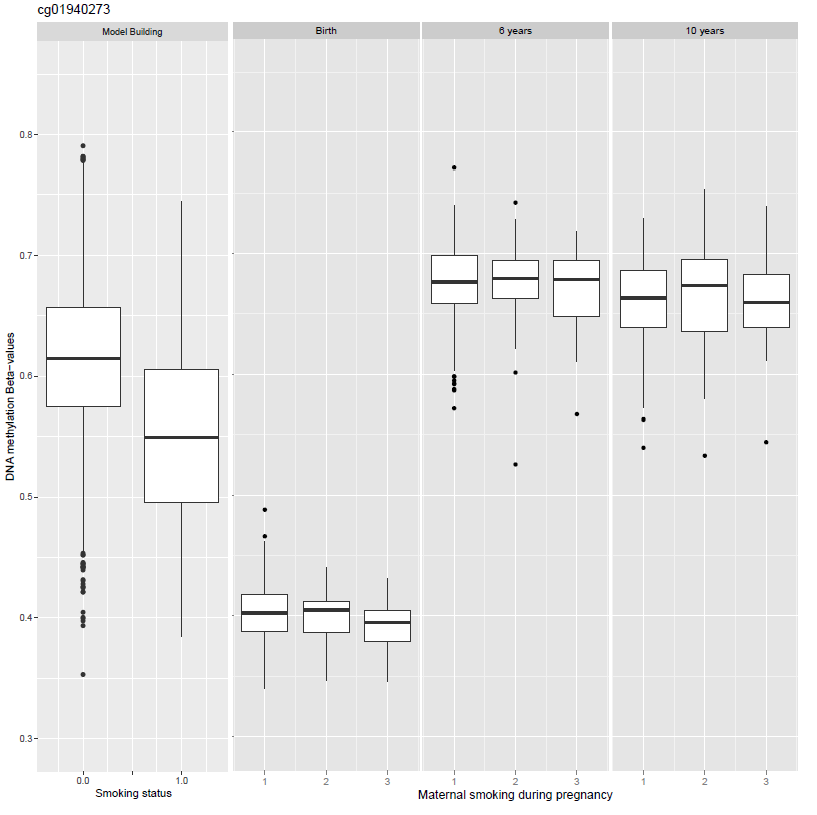 |

| **Fig. S12** β-values of cg12876356 in the model building and the Generation R Study datasets |
| --- |
| 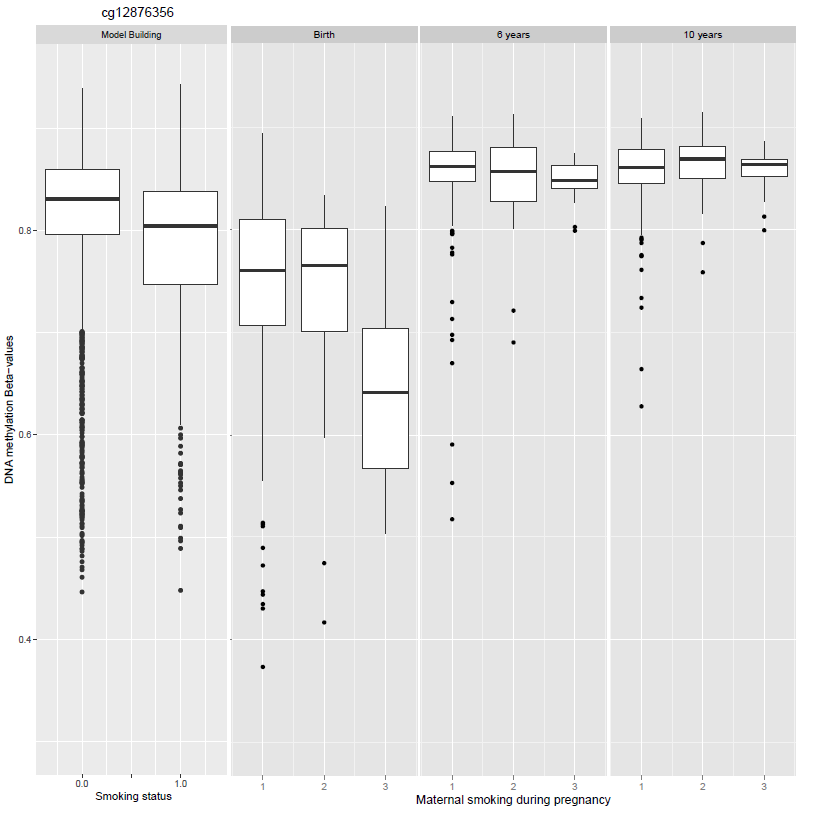 |
|  |

| **Fig. S13** β-values of cg09935388 in the model building and the Generation R Study datasets |
| --- |
| 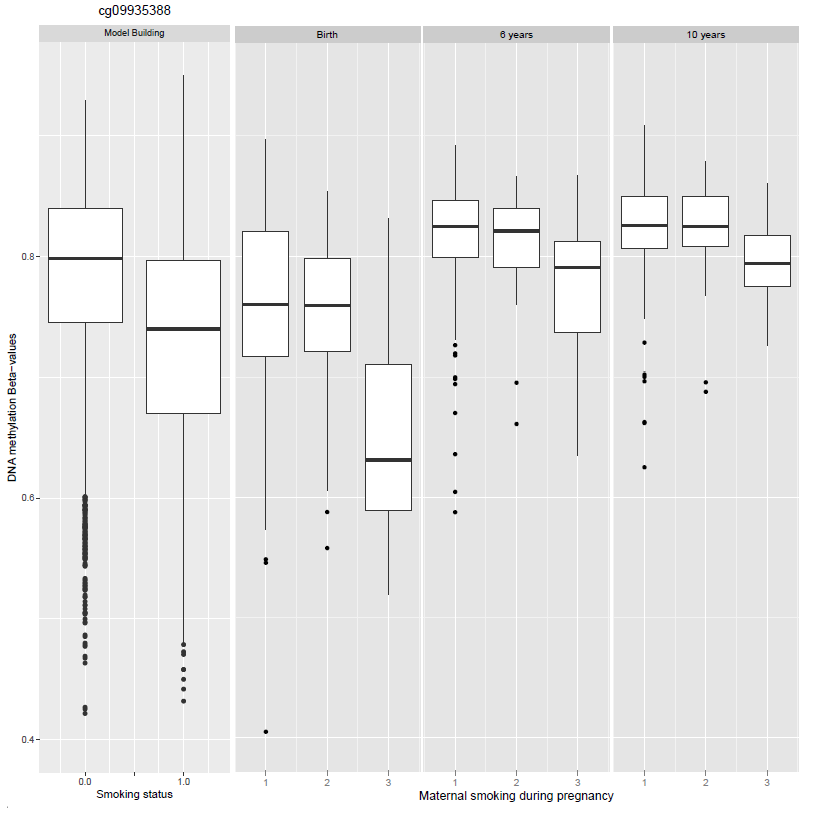 |
|  |
